# A Delphi-method-based consensus guideline for definition of treatment-resistant depression for clinical trials

**DOI:** 10.1038/s41380-021-01381-x

**Published:** 2021-12-15

**Authors:** Luca Sforzini, Courtney Worrell, Melisa Kose, Ian M. Anderson, Bruno Aouizerate, Volker Arolt, Michael Bauer, Bernhard T. Baune, Pierre Blier, Anthony J. Cleare, Philip J. Cowen, Timothy G. Dinan, Andrea Fagiolini, I. Nicol Ferrier, Ulrich Hegerl, Andrew D. Krystal, Marion Leboyer, R. Hamish McAllister-Williams, Roger S. McIntyre, Andreas Meyer-Lindenberg, Andrew H. Miller, Charles B. Nemeroff, Claus Normann, David Nutt, Stefano Pallanti, Luca Pani, Brenda W. J. H. Penninx, Alan F. Schatzberg, Richard C. Shelton, Lakshmi N. Yatham, Allan H. Young, Roland Zahn, Georgios Aislaitner, Florence Butlen-Ducuing, Christine Fletcher, Marion Haberkamp, Thomas Laughren, Fanni-Laura Mäntylä, Koen Schruers, Andrew Thomson, Gara Arteaga-Henríquez, Francesco Benedetti, Lucinda Cash-Gibson, Woo Ri Chae, Heidi De Smedt, Stefan M. Gold, Witte J. G. Hoogendijk, Valeria Jordán Mondragón, Eduard Maron, Jadwiga Martynowicz, Elisa Melloni, Christian Otte, Gabriela Perez-Fuentes, Sara Poletti, Mark E. Schmidt, Edwin van de Ketterij, Katherine Woo, Yanina Flossbach, J. Antoni Ramos-Quiroga, Adam J. Savitz, Carmine M. Pariante

**Affiliations:** 1 King’s College London, Institute of Psychiatry, Psychology and Neuroscience, Department of Psychological Medicine, London, UK; 2 Neuroscience and Psychiatry Unit, Division of Neuroscience and Experimental Psychology, School of Biological Sciences, Faculty of Biological, Medical and Human Sciences, The University of Manchester and Manchester Academic Health Sciences Centre, Manchester, UK; 3 Department of General and Academic Psychiatry, Centre Hospitalier Charles Perrens, Laboratory of Nutrition and Integrative Neurobiology (UMR INRAE 1286), University of Bordeaux, Bordeaux, France; 4 Department of Psychiatry and Psychotherapy, University of Münster, Münster, Germany; 5 Otto Creutzfeldt Center for Cognitive and Behavioral Neuroscience, University of Münster, Münster, Germany; 6 Department of Psychiatry and Psychotherapy, University Hospital Carl Gustav Carus, Medical Faculty, Technische Universität Dresden, Dresden, Germany; 7 Department of Psychiatry, Melbourne Medical School and The Florey Institute of Neuroscience and Mental Health, The University of Melbourne, Parkville, VIC Australia; 8 Mood Disorders Research Unit, The Royal’s Institute of Mental Health Research, Ottawa, ON Canada; 9 Department of Psychiatry, University of Ottawa, Ottawa, ON Canada; 10 Department of Cellular and Molecular Medicine, University of Ottawa, Ottawa, ON Canada; 11 National Institute for Health Research Mental Health Biomedical Research Centre, South London and Maudsley NHS Foundation Trust and King’s College London, London, UK; 12 Medical Sciences Division, Department of Psychiatry, University of Oxford, Oxford, UK; 13 APC Microbiome Ireland, Cork, Ireland; Department of Psychiatry and Neurobehavioral Sciences, University College Cork, Cork, Ireland; 14 Department of Molecular Medicine, Division of Psychiatry, University of Siena, Siena, Italy; 15 Translational and Clinical Research Institute, Newcastle University, Newcastle upon Tyne, UK; 16 Depression Research Center of the German Depression Foundation and Department of Psychiatry, Psychosomatics and Psychotherapy, Goethe University, Frankfurt, Germany; 17 Department of Psychiatry, University of California, San Francisco, San Francisco, CA USA; 18 Department of Psychiatry and Behavioral Sciences, Duke University School of Medicine, Durham, NC USA; 19 Université Paris Est Creteil (UPEC), AP-HP, Hôpitaux Universitaires Henri Mondor, Département Médico-Universitaire d’Addictologie et Psychiatrie (DMU IMPACT), INSERM U955, IMRB, translational Neuropsychiatry lab, Fondation FondaMental, F-94010 Creteil, France; 20 Cumbria, Northumberland, Tyne and Wear NHS Foundation Trust, Newcastle, UK; 21 Department of Psychiatry, University of Toronto, Toronto, ON Canada; 22 Department of Pharmacology and Toxicology, University of Toronto, Toronto, ON Canada; 23 Mood Disorders Psychopharmacology Unit, University Health Network, Toronto, ON Canada; 24 Brain and Cognition Discovery Foundation, Toronto, ON Canada; 25 Department of Psychiatry and Psychotherapy, Central Institute of Mental Health, Medical Faculty Mannheim, Heidelberg University, Square J5, 68159 Mannheim, Germany; 26 Department of Psychiatry and Behavioral Sciences, Emory University School of Medicine, Atlanta, GA 30322 USA; 27 Department of Psychiatry, University of Texas at Austin, Dell Medical School, Austin, TX USA; 28 Department for Psychiatry and Psychotherapy, Medical Center – University of Freiburg, Faculty of Medicine, University of Freiburg, Hauptstrasse 5, 79104 Freiburg, Germany; 29 Centre for Neuropsychopharmacology, Division of Psychiatry, Imperial College, London, London, UK; 30 Istituto di Neuroscience, University of Florence, Italy; Albert Einstein College of Medicine, New York, USA; 31 Department of Psychiatry and Behavioral Sciences, Psychiatry University of Miami, Miami, FL USA; 32 Department of Biomedical, Metabolic & Neural Sciences, University of Modena, Modena, Italy; 33 VeraSci, Durham, NC USA; 34 Department of Psychiatry, Amsterdam UMC, Vrije Universiteit and GGZinGeest, Amsterdam, the Netherlands; 35 Department of Psychiatry and Behavioral Sciences, Stanford University, Stanford, CA USA; 36 Department of Psychiatry, University of Alabama at Birmingham, Birmingham, AL USA; 37 Department of Psychiatry, University of British Columbia, Vancouver, British Columbia Canada; 38 Federal Institute for Drugs and Medical Devices (Bundesinstitut für Arzneimittel und Medizinprodukte, BfArM), Bonn, Germany; 39 Office of Therapies for Neurological and Psychiatric disorders, Human Medicines Division, European Medicines Agency, Amsterdam, the Netherlands; 40 Biostatistics, GlaxoSmithKline, London, UK; 41 Laughren Psychopharm Consulting, LLC, Rockville, MD USA; 42 GAMIAN-Europe (Global Alliance of Mental Illness Advocacy Networks-Europe), Brussels, Belgium; 43 Department of Psychiatry and Psychology, School for Mental Health and Neuroscience, EURON, Maastricht University Medical Centre, Maastricht, the Netherlands; 44 Faculty of Psychology, Center for Experimental and Learning Psychology, University of Leuven, Leuven, Belgium; 45 Data, Analytics and Methodology Taskforce, European Medicines Agency, Amsterdam, the Netherlands; 46 Department of Psychiatry, Hospital Universitari Vall d’Hebron (HUVH), Barcelona, Catalonia Spain; 47 Biomedical Network Research Centre on Mental Health (CIBERSAM), Madrid, Spain; 48 Department of Psychiatry and Forensic Medicine, Universitat Autònoma de Barcelona, Barcelona, Catalonia Spain; 49 Vita-Salute San Raffaele University, Milan, Italy; 50 Division of Neuroscience, Psychiatry and Clinical Psychobiology, IRCCS Scientific Institute Ospedale San Raffaele, Milan, Italy; 51 Strategic Projects Unit, Vall d ‘Hebron Research Institute (VHIR), Vall d’Hebron Barcelona Hospital Campus, Barcelona, Spain; 52 Charité – Universitätsmedizin Berlin, Department of Psychiatry and Psychotherapy, Campus Benjamin Franklin, Berlin, Germany; 53 Janssen Research & Development, Beerse, Belgium; 54 Charité – Universitätsmedizin Berlin, Department of Psychosomatic Medicine, Campus Benjamin Franklin, Berlin, Germany; 55 University Medical Center Hamburg-Eppendorf, Institute of Neuroimmunology and Multiple Sclerosis (INIMS), Center for Molecular Neurobiology, Hamburg, Germany; 56 Department of Psychiatry, Erasmus University Medical Centre, Rotterdam, the Netherlands; 57 Medical Director Neuroscience for Region Europe at Novartis Pharma, Basel, Switzerland; 58 Department of Psychiatry, University of Tartu, Tartu, Estonia; 59 Faculty of Medicine, Department of Medicine, Centre for Neuropsychopharmacology, Division of Brain Sciences, Imperial College London, London, UK; 60 Documental Ltd, Tallin, Estonia; West Tallinn Central Hospital, Tallinn, Estonia; 61 Department of Global Regulatory Affairs, Neuroscience, Janssen Research & Development, LLC, Titusville, NJ USA; 62 Group of Psychiatry, Mental Health and Addictions, Vall d’Hebron Research Institute (VHIR), Barcelona, Catalonia Spain; 63 Universidad Autónoma de Barcelona, Barcelona, Spain; 64 Biomedical Network Research Centre on Mental Health (CIBERSAM), Barcelona, Catalonia Spain; 65 Experimental Medicine, Janssen Research & Development, Janssen Pharmaceutica NV, Beerse, Belgium; 66 European Infrastructure for Translational Medicine (EATRIS), Amsterdam, Netherlands; 67 Neuroscience, Global Drug Development, Novartis Pharma AG, Basel, Switzerland; 68 Department of Neuroscience, Janssen Research & Development, LLC, Titusville, NJ USA

**Keywords:** Diagnostic markers, Depression

## Abstract

Criteria for treatment-resistant depression (TRD) and partially responsive depression (PRD) as subtypes of major depressive disorder (MDD) are not unequivocally defined. In the present document we used a Delphi-method-based consensus approach to define TRD and PRD and to serve as operational criteria for future clinical studies, especially if conducted for regulatory purposes. We reviewed the literature and brought together a group of international experts (including clinicians, academics, researchers, employees of pharmaceutical companies, regulatory bodies representatives, and one person with lived experience) to evaluate the state-of-the-art and main controversies regarding the current classification. We then provided recommendations on how to design clinical trials, and on how to guide research in unmet needs and knowledge gaps. This report will feed into one of the main objectives of the EUropean Patient-cEntric clinicAl tRial pLatforms, Innovative Medicines Initiative (EU-PEARL, IMI) MDD project, to design a protocol for platform trials of new medications for TRD/PRD.

## Introduction

Many medications have proven efficacy in major depressive disorder (MDD) [1, 2], but frequently and even with multiple medication exposures, they fail to improve MDD symptoms [3–7]; one third of individuals do not achieve full symptomatic remission [3], and even fewer meet criteria for both symptomatic and functional remission [8]. In individuals with ineffective initial treatments, even if subsequent treatments are effective, there is a very high relapse rate while continuing the treatment; for example, in the STAR*D trial, individuals who required more treatment steps had higher relapse rates (up to 71% after the fourth step) [3].

‘Incomplete response’ is not an all-or-nothing phenomenon, but rather a continuum that ranges from partially responsive depression (PRD), to treatment-resistant depression (TRD), to ‘multi-therapy-resistant MDD (MTR-MDD)’ [9], to ‘refractory depression’, which implies an absence of response to all currently available treatments. Unfortunately, there is a lack of consensus definitions around concepts such as PRD, TRD, and ‘adequate’ treatments [10–13]. Moreover, guidelines on how to treat TRD/PRD, such as pharmacological augmentation, are not consistent [14], and evidence on effectiveness is sparse [15]. This complicates the generalizability of results from research settings to the real-world, and hinders progress in this field, as there is no uniform population for clinical and biological investigations in TRD/PRD, including clinical trials for new or repurposed medications. Importantly, regulators acknowledge that response, partial response, and non-response exist on a continuum without universally accepted definitions, but nevertheless distinguish between these conditions; indeed, treatments for TRD and PRD already have accepted regulatory paths for drug approval.

Interestingly, a recent consensus statement (including experts also participating in the present report) suggested that the terms PRD and TRD are semantically and operationally not ideal, and proposed the broader concepts of ‘difficult-to-treat depression (DTD)’ or ‘suspected DTD’ [7], described as “depression that continues to cause significant burden despite usual treatment efforts”. This concept overlaps with PRD and TRD, but introduces a more flexible, multidimensional and longitudinal definition. The authors themselves acknowledged that “what constitutes significant burden” is “subjective and likely to vary between patients”, thus implicitly involving the patient’s point of view, and also likely to vary among clinicians and raters. Indeed, the authors cautioned that this definition may not be specific and objective enough to define clinical populations for regulatory clinical trials. In a subsequent follow-up paper, they also emphasised this as a ‘model of care’ that might be useful for both individuals with MDD and clinicians, especially in averting the development of ‘therapeutic nihilism’ [16].

In this document, we use a Delphi-method-based consensus approach to define TRD and PRD and to deliver operational criteria for future clinical studies, including clinical trials conducted for regulatory purposes. We have reviewed the relevant literature and brought together a group of international experts (including clinicians, academics, researchers, employees of pharmaceutical companies, regulatory bodies representatives, and one person with lived experience (PWLE)) to discuss the current state-of-the-art and the main controversies regarding the current classification. Our specific aims are to: (1) deliver TRD/PRD definitions that could be used at a person-centred level using information that is currently routinely collected in clinical practice; (2) recommend measures and instruments that should be included in future investigations and clinical trials for TRD/PRD; and (3) indicate which are the important areas that require further research. We provide consensus recommendations and the level of agreement for each recommendation, and discuss their limitations. We balance the need of clinicians and scientists with those of regulatory authorities, and describe differences when indicated.

This initiative is part of the EU Patient-cEntric clinicAl tRial pLatforms (EU-PEARL) programme, a public-private strategic partnership funded by the Innovative Medicines Initiative (IMI) to conceptualize and lead the design of an integrated research platform (IRP), that is, an infrastructure which allows the planning and completion of platform trials (see Appendix for further information).

## Our Delphi-method-based approach

**Fig. 1:**
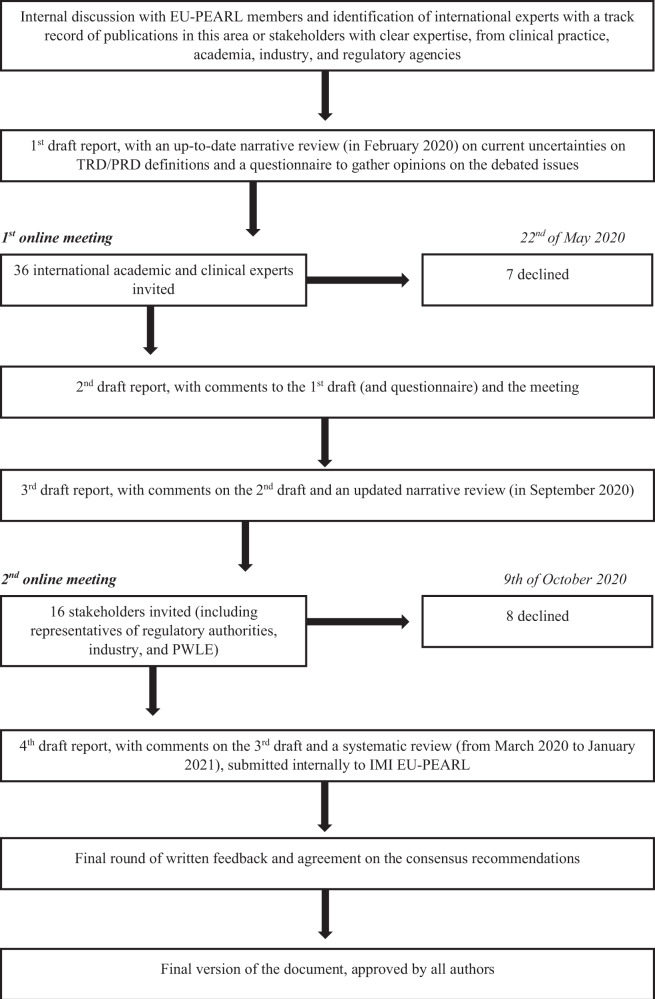
Delphi process flow diagram. Schematic representation of the different phases of the Delphi-method-based process.

The Delphi approach is a method of choice for developing guidelines in health research [17]. Indeed, a recent commentary to the aforementioned paper on DTD [7] specifically recommends using a Delphi approach for consensus statement in depression [18]. However, this method could be difficult to standardize, with poor reporting of findings [19, 20]. Probably the most important of these issues has been identified in the definition of consensus [21]. Therefore, we clearly defined consensus as a percentage of agreement (vs. disagreement) on a precise recommendation, and further defined strong consensus when this percentage was equal or above 95%, moderate consensus between 61 and 94%, and weak consensus between 51 and 60%. Details of our Delphi process are available in the Appendix and summarized in Fig. 1. Briefly, it consisted of: (1) identification of experts with a track record of publications in this area or stakeholders with clear expertise, from clinical practice, academia, industry, and regulatory agencies; (2) first draft report, with an up-to-date narrative review on TRD/PRD definitions (in February 2020) and a questionnaire to gather opinions on the debated issues; (3) an online consensus meeting with academics and clinicians, on the 22nd of May 2020; (4) second draft report, integrating comments to the first draft with those from the meeting, and again circulated to all contributors; (5) third draft report (with an updated narrative review, in September 2020), circulated to a different group of stakeholders, including representatives of regulatory authorities, industry, and one PWLE; (6) an online consensus meeting with stakeholders, on the 9th of October 2020, with additional stakeholders providing written feedback; (7) fourth draft report (with a systematic review of the literature published from March 2020 to January 2021), integrating stakeholders comments, which was then circulated and approved by all authors, and submitted in its entirety (approximately 28,000 words) internally to IMI EU-PEARL (available from the corresponding author on request); (8) editing of the report into this shorter version for publication, again circulated and approved by all authors. Comments to the different versions of the report were always anonymized. Methodological details of the narrative reviews and of the subsequent systematic review are presented in the Appendix.

**Fig. 2:**
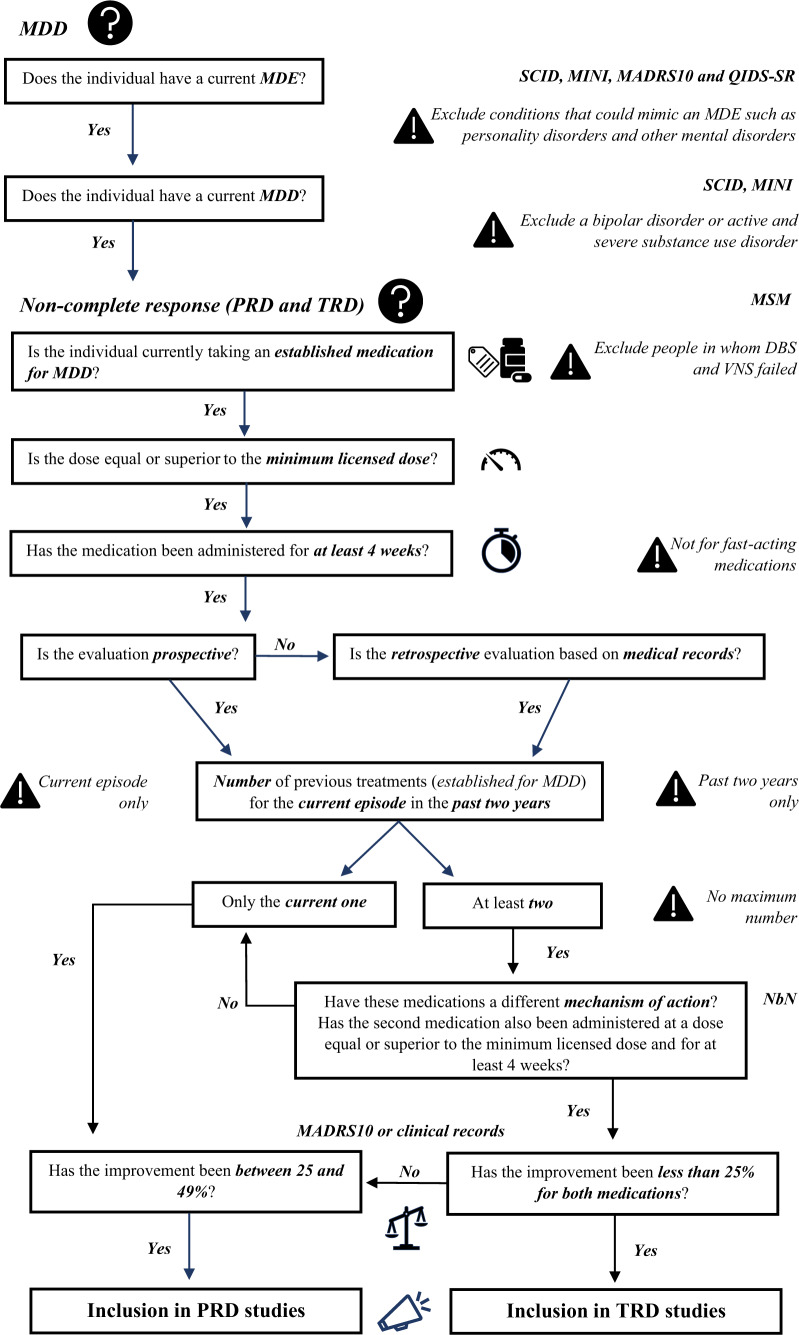

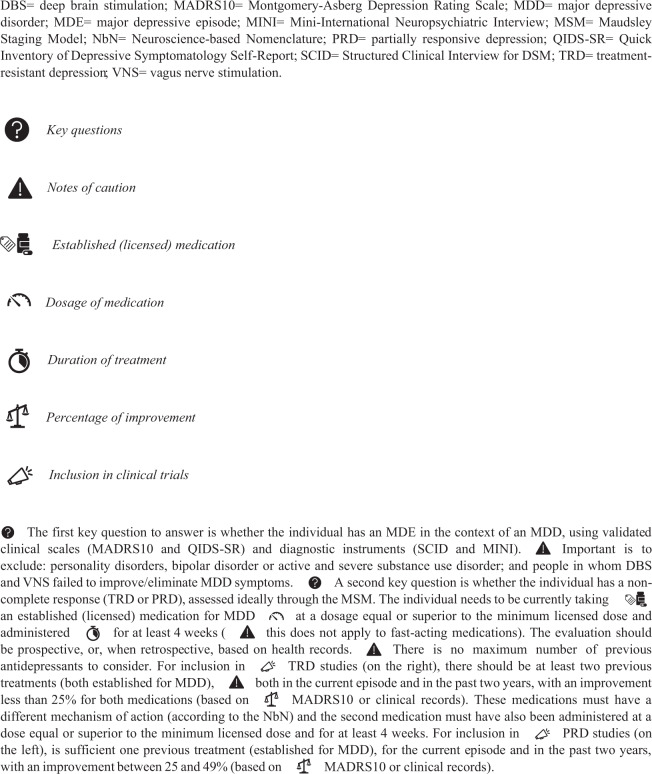
Algorithm for inclusion in TRD/PRD studies. Recommended algorithm for inclusion of participants in TRD and PRD studies based on the consensus recommendations discussed throughout the manuscript and listed in Table 1.

We provide a number of recommendations, each based on the view supported by the largest number of the experts, while highlighting areas of uncertainty and defining the ‘level of consensus’ on each recommendation: ‘strong’, indicating the top one-third of recommendations that had unanimous or almost unanimous (≥95%) agreement; ‘moderate’, indicating almost all the remaining two-thirds of recommendations, which had a substantial majority (61–94%); and ‘weak’, indicating the one recommendation that only reached the minimum consensus of 51%. We have summarised all the recommendations in Table 1 and Fig. 2. We suggest that: (1) recommendations with strong consensus should be immediately adopted; (2) recommendations with a moderate consensus could provide a working model for immediate implementation, but should be further confirmed by future research/discussion; and (3) recommendations with a weak consensus represent an area of uncertainty and thus require further research/discussion.

**Table 1:** Main consensus recommendations on TRD/PRD regulatory clinical trials.

***Level of consensus– Strong***		***% of agreement***
**Recommendations which can be implemented within current practice**		
TRD and PRD definitions		
1	A definition of TRD for clinical trials conducted for regulatory purposes is necessary.	98%
Previous antidepressant treatments		
3	TRD should be defined after a minimum of two failed treatments with <25% of improvement with adequate dosing and duration.	96%
Type of medications		
10	Discontinuation of treatment before the completion of the fourth week, without clear evidence of lack of response, should not be considered as a treatment failure for the purpose of establishing TRD/PRD.	96%
Exclusion from TRD/PRD studies		
12	A previous structured psychotherapy failing to improve MDD symptoms is not an exclusion criterion.	100%
Clinical presentation		
15	All specifiers of depression (melancholic, atypical, anxious, psychotic, mixed) should be considered within the TRD/PRD definition, except for bipolar depression.	95%
**Recommendations which can be implemented in future research**		
21	Future research should recognize and target different clinical phenotypes of TRD (and PRD) underpinned by a specific biological mechanism.	100%
22	For future research, diagnostic and history-taking instruments should be implemented in clinical cohorts and electronic health records, to allow a reliable, comprehensive, and multidimensional evaluation of people with lived experience (PWLE).	100%
24	Preferences, perspectives, and reported outcomes of PWLE should be included in future TRD (and PRD) diagnostic tools and outcome measures.	100%
***Level of consensus – Moderate***		
**Recommendations which can be implemented within current practice**		
TRD and PRD definitions		
2	It is important to distinguish between TRD and PRD for randomized clinical trials for new treatments.	85%
Previous antidepressant treatments		
4	PRD can be defined even after a single treatment (improvement 25 to <50%) with adequate dosing and duration.	76%
6	It is possible to assess ineffective past/current antidepressant treatment attempts, but only if properly documented, that is, based not only on subjective recollection or standardised instruments to assess psychiatric history and previous treatments (see below), but also on clinical documentation.	75%
Type of medications		
7	To define TRD, the two antidepressant treatment failures should consist of two established (licensed) medications for MDD of different mechanisms of action.	75%
8	A failed course of psychotherapy should not be included as one of the previous treatments required for the definition of TRD/PRD.	78%
9	The criteria of ‘adequate dose and duration’ is the minimal effective dosage, that is, the minimal approved dosage, administered for at least four weeks.	74%
Exclusion from TRD/PRD studies		
11	Multiple-drug resistant individuals, and individuals in whom augmentation strategies failed to improve/eliminate MDD symptoms should not be excluded from TRD/PRD studies.	93%
13	Individuals with MDD in whom deep brain stimulation (DBS) and vagus nerve stimulation (VNS) failed to improve/eliminate MDD symptoms should be excluded from TRD/PRD clinical studies.	62%
14	Individuals with MDD in whom other non-continuous/non-invasive brain stimulation interventions, such as electroconvulsive therapy (ECT) or transcranial magnetic stimulation (TMS), failed to improve/eliminate MDD symptoms should not be excluded.	88%
Clinical presentation		
16	Comorbid personality disorders or other mental disorders should be excluded from TRD/PRD studies only when their onset is properly documented as independent and antecedent to the MDD diagnosis.	79%
17	Individuals with a severe substance use disorder not currently in remission should be excluded from TRD/PRD studies, independently from the onset; in contrast, individuals with comorbid substance use disorder that is active and mild/moderate should be excluded from TRD/PRD studies only when the onset is properly documented as independent and antecedent to the MDD diagnosis.	83%
Diagnostic tools and measures of outcome		
18	Maudsley Staging Model is the preferred instrument to assess TRD/PRD status.	69%
19	Clinician administered MADRS10 is the preferred outcome instrument to assess treatment response (and remission), together with patient-reported QIDS-SR.	MADRS = 85% QIDS-SR = 81%
20	Criteria for remission, response, and partial response should not be relaxed in regulatory clinical trials for TRD/PRD, and shorter versions of the traditional scales, such as the HAM-D6 and the MADRS6, should not be preferred to full scales.	92%
**Recommendations which can be implemented in future research**		
23	Currently, no biomarker has been validated in clinical practice or in clinical trials to identify people with TRD (and PRD), or to further stratify them; however, collection of biological samples for subsequent subgroup or stratified analyses is recommended.	91%
25	The usefulness of adherence assessment using blood levels or other methods (also in a run-in period) should be assessed through research, before deciding whether it should be implemented in future clinical trials.	92%
***Level of consensus – Weak***		
**Recommendations which can be implemented within current practice**		
Previous antidepressant treatments		
5	The definition of TRD should include two treatment failures both within the current episode, and the definition of PRD should include partial response to at least one treatment within the current episode; moreover, for long current episodes, only treatment failures within the last two years should be considered.	51%

## TRD and PRD definitions

### What is lack of response?

A treatment response in MDD is defined by a reduction of at least 50% in MDD severity [22] on a standardised rating scale (see below); ‘lack of response’, encompassing both TRD and PRD, is the mirror image of this definition, that is, less than 50% reduction in depression severity. TRD is further associated, in most definitions, with the ‘classic criteria’ of lack of response to at least two medications at an adequate dose and duration (see below). There is, however, much variability around the definition of TRD. In a recent systematic review, Brown et al. [12] reported that, out of 155 TRD definitions identified in the published literature, 48.4% specified at least two sequential treatment failures as a requirement. In an even more recent systematic review, Gaynes and colleagues [5] found that only 37% of intervention studies in TRD had enrolled individuals with MDD meeting the criteria of at least two failed antidepressants, and only 19% had also described failure to adequate doses and durations of treatments. In fact, the most common definition for TRD in intervention trials involved a minimum of only one previous failed treatment (48%). Thus, the majority of studies on TRD do not seem to use the ‘classic criteria’, making it difficult to pool or compare data across these TRD studies. Not surprisingly, there was a strong consensus (98%) for our first recommendation that a definition of TRD for clinical trials conducted for regulatory purposes is necessary*.*

### Operational criteria for TRD and PRD

Based on a number of guidelines and other expert documents [22–25], in this report we adopt the definition of TRD as indicating individuals who show a reduction of less than 25% in MDD severity to at least two antidepressants, and of PRD as indicating individuals who show a reduction of between 25% and <50% in MDD severity to at least one antidepressant. This should ideally be established using prospective psychometric assessments, or at least using clinical interviews and health records to measure retrospectively the improvement in depression severity (see below).

The majority of experts, even though with moderate consensus (85%), recommended the importance of this distinction between TRD and PRD for randomized clinical trials for new treatments, especially because of the potential advantage of separating these individuals for different types of randomized controlled trials (for example, switching for TRD vs. augmentation for PRD).

## Previous antidepressant treatments

Here we discuss: (1) how many previous treatments should be considered, (2) in which episode (current and/or past) and (3) the way the treatment failure or partial response should be assessed for inclusion in clinical trials (prospectively and/or retrospectively).

### Number of previous treatments

The FDA guidance [26] acknowledges that no universally accepted definitions exist for TRD or PRD, and proposes that TRD studies should include people with MDD who have not responded to more than one (so, at least two) prior medications administered at an adequate dose and duration. Similarly, the EMA opts for the same definition of at least two failed treatments when considering the matter “in a clinical pragmatic view”. This same definition is confirmed as the most common in the review by Gaynes and colleagues [5], and endorsed by the DTD consensus statement by McAllister–Williams and colleagues [7], although the last document states that some individuals might be considered to have DTD even with a single treatment failure, for example when standard treatments are contraindicated. There is also an uncertainty around PRD. EMA documents do not specify a precise number of previous treatments to diagnose PRD [27], while the FDA indicates that “for adjunctive treatment, studies should include patients with partial responses to other antidepressant therapies” [26].

Consistent with the most commonly used criteria, we recommend that TRD should be defined after a minimum of two failed treatments with <25% of improvement with adequate dosing and duration (strong consensus, 96%), while PRD can be defined even after a single treatment (improvement 25 to <50%) with adequate dosing and duration (moderate consensus, 76%).

### Current or past episodes

The preferred definition of TRD for clinical trials includes a current failure and a past failure, i.e., subjects are currently receiving an antidepressant and they are still depressed according to current clinician’s assessment, and they were also treated with another antidepressant in the past and showed no response based on retrospective assessment. However, it is unclear whether both of these treatments should apply to the same (current) episode or to clearly distinct episodes. Indeed, although the EMA definition of MDD [27] emphasizes the current episode for the characterisation of the disease, it does not clarify whether the two failures should both be during the current episode. Of course, it is difficult to retrospectively defining the response to an antidepressant, especially if the current episode is of long duration (years) and the previous treatment was closer to the onset of the episode.

We recommend (with weak consensus, 51%) that the definition of TRD should include two treatment failures both within the current episode, and the definition of PRD should include partial response to at least one treatment within the current episode; moreover, for long current episodes, only treatment failures within the last two years should be considered. Of note, the “minority” position was split across a continuum of different opinions ranging from those who wanted to maintain that both antidepressants should be in the same episode but within a shorter period of time (shorter than two years) to those who wanted to consider two antidepressants “life-time”; thus, our recommendation sits in the middle of these extremes.

### Prospective or retrospective assessment

A related issue, particularly important for regulatory clinical trials, is whether one treatment failure should be ‘prospective within the trial’, i.e., the trial starts with an established medication for MDD at an adequate dose, and then the person is offered a new intervention only if the medication fails to improve or eliminate MDD symptoms. We acknowledge that this, while ideal, would lead to operational execution challenges within a trial, with increased complexity and burden for the sites and study participants [22].

We recommend (with moderate consensus, 75%) that it is possible to assess ineffective past/current antidepressant treatment attempts, but only if properly documented, that is, based not only on subjective recollection or standardised instruments to assess psychiatric history and previous treatments (see below), but also on clinical documentation, such as pharmacy, hospital, or other health records. This documentation can also be used to confirm some degree of adherence to the failed treatments, and to screen people with depressive symptoms for previous episodes of mania, hypomania, or sub-threshold bipolarity, since these individuals should be excluded (see below).

Adherence to treatment is a well-recognized critical issue in both clinical and research settings. Rates of adherence vary across the literature and generally are limited by different and/or restricted time periods [28]. However, people with MDD have typically high reported rates of non-adherence (up to more than 50%) [29, 30]. It is therefore vital to properly confirm the individual’s adherence in order to define non-response: many cases of TRD may not be true TRD, but, instead, represent partial or full non-adherence. However, the assessment of treatment adherence can be difficult and is often not addressed in everyday settings, or in most current studies in TRD. The most reliable method to assess adherence is to perform a blood test to measure the concentration of the medication in the plasma (which would also allow the recognition of fast and slow metabolisers), even though it may increase the participants’ burden during the trial. Although this suggestion was clearly supported by the experts, systematic use of plasma level monitoring in TRD/PRD definitions is not current practice. It is important to note that the FDA does not accept data analyses that exclude individuals not compliant to the previous treatment based on a blood dosing of the medicine. Further discussion on adherence is presented in the Appendix. However, we recommend assessing the usefulness of different methods to measure a person’s adherence for a potential (and desirable) future implementation (see Future directions).

## Type of medications

### Different classes and mechanisms of action

The EMA [27] mentions that the two treatment failures could be with medications of “same or different class”. Other guidelines, including the FDA guidance [26], do not make this distinction. Interestingly, the concept of ‘different mechanisms’ may overlap with ‘different classes’, although the pharmacological overlap between classes and mechanism of action is not absolute; the Neuroscience-based Nomenclature (NbN) for psychotropic agents has greatly contributed to clarify this issue [31]. Moreover, there are some geographically-relevant label limitations to the usage of some medications (see also Appendix).

We recommend (with moderate consensus, 75%) that, to define TRD, the two antidepressant treatment failures should be with two established (licensed) medications for MDD of different mechanisms of action. The two treatments could be separated by a drug free period (and there is failure to both), prescribed sequentially (switching, with failure to both), or one is prescribed as augmentation to the other (because of the first drug failing, and with the augmentation also failing).

Moreover, we also recommend (with moderate consensus, 78%) that a failed course of psychotherapy should not be included as one of the previous treatments required for the definition of TRD/PRD, but this information should be reported for staging (see below). Notably, this recommendation does not have implications for the role of psychotherapies in the treatment of TRD, and, similarly to other TRD models proposed [11], we support the use of psychotherapies as a treatment strategy for TRD in clinical practice.

### Dosage and duration of antidepressants

Most studies and meta-analyses have found no benefit for antidepressant dose escalation versus staying on the minimum licensed dose, with an increased risk of side effects and discontinuation [32, 33], even if there is also evidence that higher doses may have superior efficacy [34, 35] and that higher starting doses may be associated with higher response rates [36]. In terms of ‘minimum duration’, some studies have shown that, while two weeks can be enough to observe an initial response, a stable response/remission is usually detectable after four weeks [37, 38]. However, a complete remission may not be detectable after four weeks. For example, approximately half of remitters on citalopram in level 1 of the STAR*D trial remitted between week 6 and week 14 [39]. Of note, this is different for fast-acting medications, such as esketamine [40], but these drugs are indicated for individuals that already have TRD.

We recommend (with moderate consensus, 74%) that the criteria of ‘adequate dose and duration’ is the minimal effective dosage, that is, the minimal approved dosage, administered for at least four weeks.

Interestingly, in regulatory clinical trials, participants’ discontinuation of treatments may be considered as an endpoint equivalent to a failure [41]. However, we recommend (with strong consensus, 96%) that discontinuation of treatment before the completion of the fourth week, without clear evidence of lack of response, should not be considered as a treatment failure for the purpose of establishing TRD/PRD, especially because of the difficulty in distinguishing retrospectively between non-response and intolerance.

## Exclusion from TRD/PRD studies

Many people with lived experience (PWLE) who fulfil the proposed criteria for TRD may have tried many ineffective antidepressants (multi-drug resistance) or other (licensed or unlicensed) pharmacological augmentation interventions (see also Appendix). We discussed whether these individuals should be considered to have TRD which is ‘too severe’ for inclusion in clinical trials.

We recommend (with moderate consensus, 93%) that multiple-drug resistant individuals, and individuals in whom augmentation strategies failed to improve/eliminate MDD symptoms should not be excluded from TRD/PRD studies, provided the other criteria are met. We also recommend (with strong consensus, 100%) that a previous structured psychotherapy failing to improve MDD symptoms is not an exclusion criterion. Rather, all this information should be recorded for staging and for potential subgroup analyses.

Deep brain stimulation (DBS) and vagus nerve stimulation (VNS) are continuous treatments in which a neurostimulator (usually implanted in the chest wall) is connected to intracerebral electrodes or the left vagus nerve [42, 43]. Because of the invasiveness of these intervention, people selected for these treatments have tried an unusually large number of ineffective treatments, and thus are different from other people living with TRD [44, 45].

We recommend (with moderate consensus, 62%) that individuals with MDD in whom DBS and VNS failed to improve/eliminate MDD symptoms should be excluded from TRD/PRD clinical studies. However, we also recommend (with moderate consensus, 88%) that individuals with MDD in whom other non-continuous/non-invasive brain stimulation interventions, such as electroconvulsive therapy (ECT) or transcranial magnetic stimulation (TMS), failed to improve/eliminate MDD symptoms should not be excluded.

## Clinical presentation

### TRD and PRD symptoms

People living with MDD have a range of symptoms specifiers (for example, with melancholic features, with atypical features, with psychotic features), as well as the presence of comorbid diagnoses (for example, comorbid anxiety, bipolar depression). Potentially, some of these symptoms may be more difficult to treat compared with others, such as MDD with anxious distress [46–49].

We recommend (with strong consensus, 95%) that all specifiers of depression (melancholic, atypical, anxious, psychotic, mixed) should be considered within the TRD/PRD definition, except for bipolar depression, which should be excluded as this is part of bipolar disorder.

### Comorbidities

Individuals with a diagnosis of personality disorder (especially borderline personality disorder) frequently meet criteria for MDD, but antidepressants are unlikely to be effective and hence may mimic a non-response. Active substance users also present an increased risk of pharmacological interactions (both pharmacokinetic and pharmacodynamic), side effects, and mood symptoms due to the effects of the substance or of the withdrawal which may appear as a ‘phenocopy’ of MDD. However, it is important to highlight that these conditions are so frequently comorbid with depression that they cannot be routinely excluded, otherwise the proposed TRD/PRD definitions would not be generalisable to individuals seen in everyday clinics.

The FDA guidance also captures this tension between homogeneity and generalisability, stating that “investigators should seek demographically broad populations and avoid unnecessary restriction of study populations (e.g., by excluding patients with concomitant illness)” [26]. Indeed, the FDA does not explicitly take position on the inclusion or exclusion of personality disorders, and it explicitly encourages to consider people with a history of substance abuse, “although such inclusions should be weighed against concerns about diagnostic and medication effect confounders”, and further states that “patients whose substance use disorder is not at least in partial remission will likely be excluded from antidepressant trials depending on the level of particular confounding concerns”. On the other hand, the EMA document only broadly indicates that MDD occurring comorbid with other psychiatric disorders is not in the remit of the guideline [27].

We recommend (with moderate consensus, 79%) that comorbid personality disorders or other mental disorders should be excluded from TRD/PRD studies only when their onset is properly documented as independent and antecedent to the MDD diagnosis. Moreover, in accordance with the FDA guidance [26], we recommend (with moderate consensus, 83%) that individuals with a severe substance use disorder not currently in remission should be excluded from TRD/PRD studies, independently from the onset; in contrast, individuals with comorbid substance use disorder that is active and mild/moderate should be excluded from TRD/PRD studies only when the onset is properly documented as independent and antecedent to the MDD diagnosis.

Also, somatic comorbidities should be systematically recorded, but not excluded a priori; this includes conditions such as inflammatory, neuroendocrine, and metabolic diseases, which can influence the response to treatments [50–52].

## Diagnostic tools and measures of outcome

We also established a consensus on the best psychometric tools to measure antidepressant response (and thus TRD/PRD status and staging) both retrospectively, to improve the way we diagnose TRD (and PRD) before entering a trial, and prospectively, as tools to use in regulatory trials. A detailed summary of the different psychometric instruments is beyond this core document and is instead presented in the Appendix.

### Historical assessment of TRD/PRD status

At a minimum, assessment to define TRD should include a structured clinical interview for the diagnosis of MDD, such as the Structured Clinical Interview for DSM (SCID) [53, 54] and the Mini-International Neuropsychiatric Interview (MINI) [55], together with a scale to assess the patient’s antidepressant history, such as the Massachusetts General Hospital Antidepressant Treatment Response Questionnaire (ATRQ) [56], and the Antidepressant Treatment History Form (ATHF) [57]. A more structured method is to use staging models [6, 58], such as the Thase and Rush method [59], the Massachusetts General Hospital Staging model (MGH-s) [22], and the more recent Maudsley Staging Model (MSM) [60], which also allows the assessment of previous antidepressant treatment failure, using the Maudsley Treatment Inventory (MTI) [10].

Among the experts, there was a substantial agreement that staging models are the preferred instrument to define TRD/PRD and assess the level of treatment-resistance, even if many experts recognize the validity of the ATRQ, often accepted in regulatory clinical trials.

We recommend (with moderate consensus, 69%) the Maudsley Staging Model as the preferred instrument*.* However, both the Thase and Rush and the MGH-s models are valid alternatives.

### Assessment of depressive symptoms and response to antidepressant treatment

The presence and severity of depression are established through clinician-administered scales, such as the Hamilton Depression Rating Scale (HAM-D 17, 21, 24 items) [61], the Montgomery-Åsberg Depression Rating Scale (MADRS10) [62], and the Quick Inventory of Depressive Symptomatology (QIDS) Clinician Rating (QIDS-C) [63]; or self-reported instruments, such as the Beck Depression Inventory (BDI) [64], the Patient Health Questionnaire-9 Item (PHQ-9) [65], and the QIDS Self-Report (QIDS-SR) [63]. When possible, these scales are administered before and after a specific treatment has started, but they can also be used as a single measure to determine the current severity of the depression. Although TRD/PRD definitions centre on ‘response’, the ideal treatment goal is remission, considered as the absence of a relevant MDD symptomatology as indicated by a score of ≤7 at the HAM-D17 [66] or ≤10 at the MADRS10 [67, 68].

Of note are also the Clinical Global Impression (CGI) scales, measuring psychiatric global status [69], and the Sheehan Disability Scale (SDS), assessing functional impairment [70], which are important since even responders may continue to have significant residual symptoms and functional impairment. Indeed, people with lived experience of depression often define treatment success with an emphasis on broadly-based functional outcomes rather than remission of individual symptoms, and their perspective should be included through patient-reported outcomes (PROs) such as quality of life, wellbeing, impact of symptoms, and social and occupational functioning [71].

We recommend (with moderate consensus) that the clinician administered MADRS10 is the preferred outcome instrument to assess treatment response (and remission) (85%), together with patient-reported QIDS-SR (81%). We additionally recommend (with moderate consensus, 92%) that criteria for remission, response, and partial response should not be relaxed in regulatory clinical trials for TRD/PRD and shorter versions of the traditional scales, such as the HAM-D6 and the MADRS6, should not be preferred to full scales.

The MADRS10 and QIDS-SR were chosen based solely on experts’ feedback, and not a priori selected (see also Appendix). The MADRS10 was originally developed with the precise aim to have a scale that was more sensitive to change compared with the HAM-D17, a crucial feature for clinical trials [72]. In addition, unlike the HAM-D [73], it predominately focuses on the core symptoms of depression such as sadness, tension, lassitude, pessimistic thoughts, and suicidal thoughts. This results in a higher internal consistency and a greater accuracy for the MADRS compared with HAM-D [74]. However, this is also a potential limitation of the MADRS, as it may not capture the clinical complexity of the PWLE. Most notably, individuals with MDD with non-melancholic or atypical features may present with a range of symptoms that are not captured by the MADRS. For such reason, there is a risk that MDD individuals continuing to report depressive symptoms such as irritability and anxiety may be classified as responders. The same issue may arise with a ‘short’ scale such as the QIDS-SR, which again carries the risk of omitting the measure of MDD symptoms that are not captured by standard diagnostic criteria of MDD, in contrast to far more granular self-report measures of depressive symptoms, such as the Inventory of Depressive Symptomatology (IDS)-SR [75], and the Symptoms of Depression Questionnaire (SDQ) [76]. Thus, the assessment of changes in MDD severity (upon which the TRD/PRD definitions rely) should include the entirety of symptoms that an individual displays, including different (non-melancholic) MDD specifiers and symptoms not included in standard classification systems. When possible, the broadest assessment possible should be conducted, using broad-spectrum scales or checklists assessing a wide range of MDD related symptoms, such as the HAM-D28. Also, specific instruments assessing specific symptoms, such as anhedonia, motor retardation, or anxiety (for example, the Hamilton Anxiety Rating Scale (HAM-A) [77]), may be useful. Sub-analyses on specific clusters of symptoms (such as suicidal, atypical, or psychotic symptoms) could be used to generate hypotheses for future clinical trials, for medications targeted to specific symptoms or to symptoms-based subgroups of individuals. Another issue is that traditional scales address depressive symptoms in the emotional and physical clusters, with a lower attention on the cognitive ones, which may frequently remain as residual symptoms indicating poor response and thus an increased risk for relapse. Interestingly, there is evidence that a mismatch between scores in clinician-administered and self-reported scales is a poor prognostic sign [78], supporting the position endorsed in this report to have at least one score of both types of scales included, until new and more tailored instruments will be developed (see also next section and Appendix).

## Future directions

This section makes recommendations that are not immediately applicable to clinical trials and highlights research gaps and new opportunities identified throughout the consultative process. Future trial sponsors may wish to incorporate one or more of these areas to expand the knowledge base. A more detailed discussion of these points is presented in the Appendix; here, we want to emphasise the main recommendations:

- Future research should recognize and target different clinical phenotypes of TRD (and PRD) underpinned by a specific biological mechanism (strong, 100%).
- For future research, diagnostic and history-taking instruments should be implemented in clinical cohorts and electronic health records, to allow a reliable, comprehensive, and multidimensional evaluation of the PWLE (strong, 100%).
- Currently, no biomarker has been validated in clinical practice or in clinical trials to identify subjects with TRD (and PRD), or to further stratify them; however, collection of biological samples for subsequent subgroup or stratified analyses is recommended (moderate, 91%).
- Preferences, perspectives, and reported outcomes of PWLE should be included in future TRD (and PRD) diagnostic tools and outcome measures (strong, 100%).
- The usefulness of adherence assessment using blood levels or other methods (also in a run-in period) should be assessed through research, before deciding whether it should be implemented in future clinical trials (moderate, 92%).

## Limitations and conclusions

While we discuss many points of debate in the Appendix, here we want to acknowledge the main limitations of this report.

First, the recommendations are based on experts’ and stakeholders’ clinical and professional judgement, supported by research and clinical evidence, but without any hard and objective validation. Second, our recommendations try to strike a balance between being ‘too broad’ and being ‘too narrow’, with the aim to identify ‘clinically homogenous’ samples, based on a pragmatic and non-aetiologically-based approach; still, we cannot exclude that significant phenotypic or biological heterogeneity is present within the defined TRD/PRD samples, and future research will allow to define this inherent variability, if any, in clinical features, biomarkers, and clinical response. Third, the opposite risk is also present: that we define groups so narrowly that the findings are not generalisable to the larger population of PWLE in clinical settings, and do not translate into better care; however, maintaining narrow definitions may help avoiding overmedicalisation and may prevent the lowering of the threshold above which treatments which are specific for TRD are offered to patients, as a recent commentary on this topic highlighted [18].

In terms of theoretical limitations, some of our contributors have argued that the proposed TRD/PRD definitions are unhelpful from a clinical and a conceptual perspective, as they arbitrarily apply thresholds on a continuum, and are influenced by the different healthcare systems. Moreover, TRD and PRD concepts are based upon a conceptualisation of depression as an episodic illness with good inter-episode recovery, while many individuals with MDD have a chronic illness with waxing and waning course; this might create difficulties when trying to accurately identify the beginning and end of the current/previous episodes, in order to define response to the antidepressants. Finally, we recognise that, according to our recommendations, individuals with MDD with an ineffective (<25%) single antidepressant trial are not defined as either TRD or PRD; as in standard clinical practice, these subjects should be prescribed a second antidepressant before a decision can be made on their TRD status.

Notwithstanding these limitations (and the points of uncertainties further discussed in the Appendix), this report offers clear and consistent definitions of TRD/PRD for regulatory clinical trials and for clinical and biological studies more broadly, agreed among a large group of experts including clinicians, academics, industry, regulatory agencies, and PWLE. Our ultimate ambition is to advance tailored treatments and a truly ‘precision medicine’ approach for MDD, which in turn will finally help to deliver better care for people suffering from this severely challenging illness, which remains too often ineffectively treated.

## Disclaimer

This document reflects the majority views of the experts. Authorship reflects having been part of the process and accepting the consensus statements without necessarily personally endorsing every single recommendation (see level of consensus). Also, the views expressed in this article are the personal views of the author(s) and may not be understood or quoted as being made on behalf of or reflecting the position of the regulatory agency/agencies or organisations with which the author(s) is/are employed/affiliated. This publication reflects only the authors’ view, and the JU is not responsible for any use that may be made of the information it contains.

## Supplementary information

Appendix
