# Supplementary material for: A Delphi-method-based consensus guideline for definition of treatment-resistant depression for clinical trials
Source: Mol Psychiatry. 2021 Dec 15;27(3):1286–99. doi: 10.1038/s41380-021-01381-x (PMC9095475; doi:10.1038/s41380-021-01381-x)
Supplement: Supplementary file 1 — Appendix [file 41380_2021_1381_MOESM1_ESM.docx]

*For resubmission to Molecular Psychiatry*

**A Delphi-method-based consensus guideline for definition of Treatment-Resistant Depression for clinical trials**

**Appendix**

**Table of Contents**

[**The EU-PEARL project** 3](#_Toc78804753)

[**Delphi process for this report** 4](#_Toc78804754)

[**Review of the literature** 5](#_Toc78804755)

[**Background on psychometric instruments for historical assessment of TRD/PRD status** 7](#_Toc78804756)

[**Future directions for research in TRD/PRD** 9](#_Toc78804757)

[**Clinical phenotypes of TRD and PRD and dimensional approach** 10](#_Toc78804758)

[**Genetics and biological markers** 11](#_Toc78804759)

[**Preferences and perspectives of PWLE** 12](#_Toc78804760)

[**Adherence** 14](#_Toc78804761)

[**Supplementary Discussion** 14](#_Toc78804762)

[**TRD and PRD definitions** 14](#_Toc78804763)

[**Previous antidepressant treatments** 15](#_Toc78804764)

[**Type of medications** 17](#_Toc78804765)

[**Exclusion from TRD/PRD studies** 19](#_Toc78804766)

[**Clinical presentation** 21](#_Toc78804767)

[**Diagnostic tools and measures of outcome** 22](#_Toc78804768)

[**References** 24](#_Toc78804769)

[***Table S1:*** Table of contributors 33](#_Toc78804770)

[***Table S2:*** CREDES checklist of the Delphi process 35](#_Toc78804771)

[***Figure S1:*** Systematic review flow diagram 37](#_Toc78804772)

[***Supplementary File S1:*** 1^st^ meeting agenda 38](#_Toc78804773)

[***Supplementary File S2:*** 2^nd^ meeting agenda 41](#_Toc78804774)

[***Supplementary File S3:*** Introductory report 42](#_Toc78804775)

# **The EU-PEARL project**

This initiative is part of a broader European project, the EU Patient-cEntric clinicAl tRial pLatforms (EU-PEARL). This is a public-private strategic partnership funded by the Innovative Medicines Initiative (IMI) to conceptualize and lead the design of an integrated research platform (IRP), that is, an infrastructure which allows the planning and completion of platform trials, through a network of investigational sites, with a federated and shared data platform and agreed regulatory pathway. The overall program will focus on four different diseases: major depressive disorder (MDD), tuberculosis, non-alcoholic steatohepatitis, and neurofibromatosis. Thirty-six institutions, including European university hospitals, research centres, people with lived experience (PWLE) groups, non-profit product developers and pharmaceutical companies, are cooperating in this consortium. The final objective is to shape future medication development through a systematic approach that enables cross-company collaborative platform trials that are PWLE-driven by design and PWLE-centred by outcome. One work package specifically concentrates on MDD, particularly for individuals in whom first-line antidepressant treatment(s) failed to improve or eliminate MDD symptoms, and this report has been produced as part of this work package. More information on the project can be found on the official website: <http://eu-pearl.eu>.

Platform trials are clinical trials with a single master protocol in which multiple treatments are evaluated simultaneously and/or sequentially^1^,^2^. Importantly, they can have an adaptive design – with flexible features – allowing the removal of treatments or groups (for example, for futility) or the addition of new groups or treatments during the course of the trial^1^,^3^. Since there are many medications for the first-line treatment of MDD^4^, EU-PEARL focuses on individuals who do not respond adequately to initial treatment(s) (treatment-resistant depression, TRD, and partially responsive depression, PRD), with the aim of developing methodology for clinical studies on people living with TRD and PRD, especially for randomized clinical trials, including clinical trials for new or repurposed medications. EU-PEARL project will, for the first time, allow adaptive platform trials to focus on TRD/PRD. In addition to developing an IRP for MDD, the MDD work package of EU-PEARL also aims to develop the protocol for a longitudinal natural history study, to better understand the course of MDD as well as to develop a prospectively-identified and recruitment-ready cohort for IRP studies.

# **Delphi process for this report**

In order to gather experts’ opinion on these gaps in knowledge, we invited experts with a track record of publications in this area or stakeholders with clear expertise, from a broad range of specialties and diverse experiences in clinical practice, academia, industry, and regulatory agencies, including clinicians and scientists associated with the EU-PEARL project. All the contributors and their areas of expertise are listed in *Table S1*. As a first step, we developed a draft document on the up-to-date literature on current TRD/PRD definitions and inclusion/exclusion criteria for regulatory clinical trials and for clinical trials for new treatments in these indications (narrative review, *see below*), with a questionnaire at the end to gather opinions on the most debated issues. After we received experts’ comments and answers, we then organized a first consensus meeting by video conference, conducted on the 22^nd^ of May 2020*,* with international experts and EU-PEARL members. Here, the experts gave presentations focused on key points of uncertainty. Each session was followed by a discussion within the whole group about the requirements for future regulatory studies. We merged all the comments and answers to the first draft with those arisen from the meeting; the resulting second draft was circulated again to all the contributors for their feedback. The resulting third draft (with an updated narrative review) was circulated to a new group of stakeholders, including representatives of regulatory authorities, industry, and one PWLE, and discussed in a second online consensus meeting on the 9^th^ of October 2020, with stakeholders and EU-PEARL members, with additional stakeholders providing written feedback. A fourth version (with a systematic review) was then circulated and approved by all authors, and submitted in its entirety (approximately 28,000 words) to IMI EU-PEARL as a milestone (available from the corresponding author on request). The report was then edited into this final shorter version for publication, again circulated and approved by all authors. Contributors’ identity was revealed, also to allow face-to-face meetings, however all comments to the different versions of the report were anonymized.

# **Review of the literature**

In order to identify the areas of uncertainties to be discussed in this Delphi report, and to prepare the “Introductory report” with the original questionnaire sent to the experts for the initial anonymous response (*Supplementary File S3*), we performed a comprehensive, not-systematic narrative review of recent systematic reviews, consensus papers, and regulatory documents on TRD/PRD, up to the end of February 2020. This material was elicited through searches in PubMed and websites of regulatory organisations, and integrated with key references identified by the EU-PEARL experts that prepared the “Introductory report”, as well as references cited by the initial pool of documents. An update of the narrative search was conducted in September 2020 for the third draft report and the stakeholders meeting, and a final systematic review was performed for the completion of the report in January 2021.

We conducted an initial not-systematic narrative review in February 2020. Using MEDLINE PubMed® we searched for the following MeSH terms and keywords: (“treatment-resistant depression” or TRD), (“partially responsive depression” or PRD), and (“major depressive disorder” or MDD or “unipolar depression” and “response to treatment”). We have further filtered results including only systematic reviews, meta-analyses and documents published in the last ten years. In addition, we searched for consensus statements, management guidelines, and regulatory documents through other websites and platforms, including British Association for Psychopharmacology (BAP), European Medicines Agency (EMA), Food and Drug Administration (FDA), National Institute for Health and Care Excellence (NICE), National Institutes of Health (NIH), and World Federation of Societies of Biological Psychiatry (WFSBP). In September 2020, we performed an updated search using the same criteria. We then summarized evidence from the most recent papers relevant to our discussion. These included the reviews by Gaynes et al.^5^, Salloum and Papakostas^6^ and McAllister-Williams and colleagues^7^, as well as the recent consensus statement by McAllister-Williams et al.^8^. In particular, the review by Gaynes et al.^5^ comprises 185 unique studies in TRD; the one by Salloum and Papakostas^6^ discusses 18 articles with proposed TRD definition and staging model, and empirical work to support the model/definition; McAllister-Williams et al.^7^ discuss a concept of multi-therapy resistant (MTR)-MDD, defining PWLE well in excess of a TRD definition and representing a potential ‘upper limit’ for inclusion in this kind of studies; and the consensus statement (McAllister-Williams et al.)^8^ discusses the concept of difficult-to-treat depression (DTD). Key documents from regulatory authorities included the FDA draft guidance^9^ and the EMA guideline^10^ on depression.

To complete the final version of the document, before final approval of all the contributors, we performed a systematic review, with the aim to identify any relevant paper published in the last few months (from March 2020 to January 2021). The flow diagram of the systematic search is reported in *Figure S1*. Using MEDLINE PubMed® database, we systematically searched for reviews, systematic reviews, meta-analyses, and guidelines published from the 1^st^ of March 2020 to the 22^nd^ of January 2021, using the following search string: ((Treatment-resistant depression) or (TRD) or (partially responsive depression) or (PRD) or (difficult-to-treat depression) or (DTD)) and ((definition) or (diagnosis) or (criteria)). The electronic search returned 50 records, 20 of which specifically evaluating individuals with non-responsive MDD. Only one article – besides the aforementioned statement by McAllister-Williams et al.^8^ – provided a rationale for TRD or PRD definitions^11^. In this review, Jackson et al. stressed the importance of distinguishing between inadequate response (PRD) and non-response (TRD), presenting the same definitions and the same cut-offs we propose in our document, further corroborating our recommendations^11^.

# **Background on psychometric instruments for historical assessment of TRD/PRD status**

The minimum required approach to assess treatment resistance consists of using structured clinical interviews for the diagnosis of MDD, such as the Structured Clinical Interview for DSM (SCID)^12^,^13^ and the Mini-International Neuropsychiatric Interview (MINI)^14^, together with specific scales to assess the patient's antidepressant treatment history, such as the ATRQ (Massachusetts General Hospital Antidepressant Treatment Response Questionnaire)^15^, and the Antidepressant Treatment History Form (ATHF)^16^. The essential value of the structured interviews is to confirm the diagnosis of MDD and to assess the presence of TRD throughout the individual’s entire clinical history, also allowing the identification of comorbidities. The antidepressant treatment history scales, ATRQ and ATHF, examine the previous antidepressant treatments as told by the person, and both have pros and cons: the ATRQ is easier to use for both patients and clinicians, but includes only the subjective recollection of how much they improved on any antidepressant trial^17^; the ATHF has the advantage of integrating clinical judgement in evaluating the adequacy of a treatment, but is quite long, and thus has been recently revised and updated in a short form (ATHF-SF)^18^.

A highly more structured method is to use staging models^19^,^6^. Probably the most commonly used model is the Thase and Rush method^20^, with TRD (as described in this report) being equivalent to stage 2 of this model, which considers the “failure of at least 2 adequate trials of medications (of different classes)”. Despite its wide use, the Thase and Rush approach has some limitations, including the absence of a clear definition of what is an appropriate antidepressant trial in terms of dosing and duration, with the risk of also including interventions which did not have the opportunity to show an effect because of either too low of a dosage or too short of a duration, or both. Furthermore, people with MDD do not necessarily progress in a linear way through the proposed hierarchy of medications (SSRIs<TCAs<MAOIs), so more treatment-resistant individuals may not necessarily reach higher stages of their scheme. Moreover, individuals who were unresponsive to TCAs were shown to benefit from a switch to SSRIs^21^,^22^. Finally, there is no consideration of augmentation strategies and psychotherapy. Another staging model is the Massachusetts General Hospital Staging model (MGH-s)^23^. This model defines TRD as the “failure to respond to at least one antidepressant trial of standard doses lasting 6 weeks or more”, so it is more permissive than the EMA and FDA criteria as well as the consensus recommendation proposed in this report in terms of number of treatments, albeit more restrictive in duration. This model generates a continuous score, with different points attributed to different clinical features, reflecting the total level of resistance. Interestingly, this scale includes “optimization of dose and duration, augmentation or combination options”, as additional steps beyond the adequate trial, attributing the same score to each of these steps. A third, more recent tool, is the Maudsley Staging Model (MSM)^24^. As with the MGH-s, the MSM also considers one antidepressant treatment failure as enough to define TRD; however, it supports the notion that TRD exists as a continuum, further elaborating that “failure of the first treatment is influential in treatment resistance and may be a useful starting point in any measure of this conceptual continuum”^6^. The MSM has been proven to have a very high predictive utility for future treatment response^19^,^6^; it can be used both as a continuous score (from 3 to 15) and divided into three ordinal categories (mild: 3-6, moderate: 7-10, severe: 11-15). The MSM also allows a ‘dimensional’ staging, based on duration, severity, and treatment, and it is also predictive in non-antidepressant-treated individuals, being a more general indicator of duration, chronicity, and severity, beyond treatment response alone^25^. Finally, the MSM also allows the assessment of previous antidepressant treatment failure, using the Maudsley Treatment Inventory (MTI), which is a new tool to assess a person’s antidepressant treatment history developed for the purposes of completing the MSM^26^. As a potential limitation, MSM arbitrarily divides the duration of illness into three categories (acute: ≤12 months, sub-acute: 12-24 months, chronic: >24 months), giving a ‘higher score’ for TRD for longer duration, irrespective of the treatment history, while one could argue that longer duration of illness is not necessarily an index of treatment-resistance. A recent staging system proposed by Conway et al.^27^ identified as TRD the failure of two different adequate dose-duration and established treatments for MDD, and thus confirming the most commonly used criteria described in literature and supported in our paper.

# **Future directions for research in TRD/PRD**

This section makes recommendations that are not immediately applicable to clinical trials but should be addressed by collecting new data as part of clinical research, longitudinal cohorts, or electronic health records.

## **Clinical phenotypes of TRD and PRD and dimensional approach**

It is essential for MDD research to move away from the idea that all PWLE are the same. Even though it is compellingly clear from the clinical picture, and response to treatments, that MDD is *not* as a single entity, this is the view commonly endorsed by clinicians, pharmaceutical companies, and regulatory agencies. In contrast, targeting treatments to ‘biologically-based subgroups of individuals with MDD’ can bring ‘personalised medicine’ to psychiatry, akin to what has happened in oncology where cancer types are treated more in relation to their biological and molecular features than to their diagnostic classification. In MDD, genetic factors are far more complex than in cancer, with a myriad of risk genes rather than few genes of relatively large effect. However, we could be focusing on subgroups of people with MDD based on specific clusters of symptoms, especially if mediated by a specific biological abnormality. This concept is also valid from a regulatory perspective and for the development of trial platforms; future trials may aim to enrich for symptom domains based on the medication to be tested. In order to do this, we need research using full scales assessing a broad range of depressive symptoms (such as the HAM-D28), or scales assessing specific symptoms that may have an underlying biological mechanism, such as anhedonia, putatively linked to inflammation and dopamine deficit^28^. These would also allow research in specific symptoms that remain *after treatment,* and thus in the development of adjunctive therapies targeting residual depressive symptoms. In addition, new digital applications for monitoring individuals’ symptoms, mainly as self-assessment instruments, may be implemented for future research.

Another approach to be examined in future research is the use of a ‘continuum’ approach in defining treatment-resistance. For example, in hypertension there is a clear definition for treatment-resistant hypertension, that is, resistance to maximal doses of 3 antihypertensives of different classes. This is different from other categories but on a continuum of severity of resistance which progresses to controlled resistant hypertension (blood pressure controlled on 4 or more antihypertensives) and then to refractory hypertension (blood pression uncontrolled despite maximal doses of 5 or more antihypertensives of different classes)^29^. Interestingly, in treatment-resistant hypertension the therapeutic approach is multidimensional, with a strong emphasis not only on clinical data (symptoms and treatments), but also on risk factors, demographic data, and lifestyle factors, mainly diet and physical exercise – something which has not been tested coherently in TRD yet, but could be extremely important in the future.

## **Genetics and biological markers**

All the experts agreed that biomarkers are extremely important for future research, but a consensus emerged that there are no genetic/biological markers currently ready to use for inclusion/exclusion of people with TRD/PRD in clinical trials. However, several biomarkers held promise for future research, especially as part of more complex diagnostic and predictive algorithms, which include clinical, cognitive, blood based, genetic, and neuroimaging markers.

Several genetic markers of treatment response in MDD have been explored^30^. The experts highlighted cytochrome P450 polymorphisms, especially of the enzymes CYP2D6 and CYP2C19^31^, which could affect an individual’s metabolism of different compounds. Also, biomarkers related to immune function were discussed as markers of TRD, including gene expression signatures^32^,^33^, cellular immunophenotype^34^, and soluble factors such as C-reactive protein (CRP)^35^,^36^. Other candidate markers include genetic profile or expression levels of biomarkers of HPA axis activity^37^,^38^; channels controlling efflux of drugs from brain, e.g., ABCB-1^39^; serotonin transporter promoter^40^; serotonin 1A or 2A receptors^41^,^42^; olfactomedin-4^43^; and brain-derived neurotrophic factor (BDNF) gene^42^. Even though we recognize the validity of these encouraging findings, we support the need for further research before the recommendations of specific biomarkers for inclusion in the regulatory trials. With this in mind, of course we advocate that current and future clinical trials at least collect the biological samples needed for subsequent testing of candidate biomarkers (DNA, whole blood mRNA, serum or plasma), or candidate biomarkers that are clearly related to the drug’s mechanisms of action (for example, an immune biomarker for an anti-inflammatory drug), so that post-hoc analyses on sub/stratified samples are possible, and can inform future confirmatory trials. Interestingly, the development of biomarkers for subtypes of depression could be used to improve our ability to eliminate earlier, and at lower costs, treatment targets that are not ultimately going to pan out, an approach referred to as ‘Fast-Fail’^44^.

## **Preferences and perspectives of PWLE**

There was a substantial agreement on the value of including preferences of PWLE when evaluating treatment resistance. This is another important point which could help to move MDD research away from a one-size-fits-all approach. Indeed, there was a general consensus on the importance of looking at TRD and PRD from a PWLE perspective, thus not only including individuals’ preferences, but also integrating them into the definition, together with their subjective experience, to better understand this condition. To develop this report, we involved PWLE representatives in the stakeholders meeting and feedback, to fully consider their viewpoint.

An important message that came out of PWLE is that clinical scales often do not adequately consider the PWLE perspective, with a consequent potential discrepancy between clinician- and patient-reported outcomes (PROs)^45^. The key example of this is the fact that a considerable proportion of people with remitted MDD (remitted according to the traditional clinical scales) do not consider themselves as remitted, because of the persistence of specific symptoms not adequately recorded, such as persistent residual cognitive symptoms or poor functionality, which obviously affect the outcome^46^. As mentioned above, this discussion is relevant for the development of future protocols to assess individuals with MDD longitudinally and to improve the future clinical trials for TRD/PRD, both of which are key aims of this IMI European Union research programme. Indeed, various approaches have been suggested to include PWLE preferences and perspectives in TRD/PRD definitions, including the importance of recording which symptoms are the most disabling for the individual and what are the relevant functional outcomes, which may be more important than purely symptomatic ones. Some experts suggested the use of specific scales, such as quality of life (QoL) scales, specific checklists, or a visual analogue scale (VAS). Because there is no evidence of clear superiority of a single instrument over others, we do not recommend the use of any specific scale. It was also discussed whether PROs should be included, as opposed to being reported as a secondary outcome measure in trials, in the definition of TRD and PRD. The recommendation is to integrate PWLE preferences and perspectives in future studies, as also clearly highlighted by stakeholders. While we endorse the use of self-reported measures of depression, such as QIDS-SR, the feedback from the stakeholder clearly indicated that these instruments, whilst offering a subjective viewpoint, still do not provide a comprehensive assessment of the individual’s preferences, perspectives, and reported outcomes, which should be included in a standardized manner in future protocols. Ultimately, future research should better identify the outcomes which are important to PWLE and consider them to better define what clinical measures to prioritise. For example, it is important to understand the individual’s target, whether it is a return to premorbid status or reaching an ‘optimal’ functioning level. Setting the bar too high could be counterproductive for PWLE themselves, potentially masking an effective treatment.

## **Adherence**

Besides blood testing of the plasma levels of medications, experts also suggested the potential use of other specific methods to assess treatment adherence in clinical trials. These could, for example, detect specific markers, such as odorous compounds or olfactory markers, upon breath exhalation after medication is taken (Xhale®), or use of digital applications, such as diaries and reminders, or artificial intelligence to match the individuals with their medications and verify intake (AiCure®). Within the authors of the present report, some maintained that ensuring minimal compliance, estimating plasma penetration, and verifying adequacy of the dispensing, is absolutely essential. Therefore, in their opinion, assessing participant’s adherence, by at least a plasma sampling at estimated steady-state and at the end of the study should be mandatory. However, the majority felt that, currently, such a recommendation for mandatory implementation is not possible. Finally, future research should assess whether it may be useful to measure compliance in the run-in period, prior to the clinical trial is initiated, to screen out non-compliant participants^47^. This could be integrated into future protocols for regulatory purposes, to guarantee the reliability of findings.

# **Supplementary Discussion**

## **TRD and PRD definitions**

A number of theoretical and practical issues were raised when discussing both the overall approach of this report and the individual recommendations, and it is important to acknowledge that, while all recommendations express the majority opinion, most only reached “moderate” consensus, and one reached “weak” consensus (that is, barely the majority).

Some of our contributors have argued that the proposed definitions of TRD and PRD are unhelpful from a clinical and a conceptual perspective, as they arbitrarily apply thresholds on a continuum, and would be influenced by different healthcare systems. Also, having consolidated definitions with strict criteria may create a hurdle for participants’ recruitment in TRD/PRD studies, and the selection of individuals that are not representative of common clinical practice. Other documents discuss this important issue. The EMA guideline highlights the discrepancy between everyday practice and the conceptual elaboration and definition of clear criteria for TRD and PRD while recommending the generation of validated criteria to address this^10^ – as indeed we do in this report. It is debatable whether two distinct definitions are needed, or a single category, or a dimensional, ‘staging’ approach, on the continuum between response, PRD, TRD, MTR-MDD, and refractory depression. Of course, the regulatory implications of differentiating between TRD and PRD are quite clear, as the stricter TRD definition could be used to enrol participants in randomized controlled trials for new MDD medications, while people with PRD may be preferentially recruited for trials to study augmentation/add-on treatments^10^,^48^,^9^. However, this ‘design-based’ distinction is not entirely supported from literature evidence. As an example, in a pooled analysis of two studies examining the efficacy of adjunctive aripiprazole in MDD, individuals with TRD seemed to have a larger treatment effect compared with those with PRD^49^; and, vice versa, treatment with intranasal esketamine has been proven effective in addition to standard of care antidepressant therapy in individuals with TRD^50^,^51^.

## **Previous antidepressant treatments**

We recognise that the number of previous treatments required to define TRD and PRD is an arbitrary decision, but we all agree on the need of such decision in order to standardise definitions of such samples. This would also reduce the influence of the level of care where the recruitment takes place; for example, in real-world clinical practice, it is more likely that a person in primary care settings would be considered as resistant after one single treatment, while it is more common to consider the resistance after the second or third treatment in secondary care, where individuals are seen after they have received the first treatment in primary care.

Concerns were also expressed on the difficulty in distinguishing a ‘relapse’ (the return of MDD symptoms after remission, but before recovery) from a ‘recurrence’ (the onset of a new episode following recovery)^52^. Indeed, it is often problematic to correctly differentiate between true ‘treatment-resistance’ and other conditions, such as ‘tachyphylaxis’ (the loss of treatment efficacy after a transient improvement)^53^, ‘pseudo-resistance’ (due to inappropriate or inadequate treatments, like suboptimal prescriptions, poor adherence)^54^, or illness relapses and recurrences following successful response^55^.

We recommend that only treatments in the last two years of the current episode can be reliably assessed. Yet, some experts suggested that only the past one year can be reliably assessed retrospectively, while others suggested that up to 5 years could be assessed, and yet others suggested to use a generic definition of ‘only for a period when the assessor is convinced that adequate and clear evidence is available’. A number of apparent incongruities were highlighted based on different definitions. For example, with a 5-year window, people would be considered TRD if they had experienced depression for 5 years and took one medication 5 years ago with no response, and a second medication currently, also with no response. However, with the recommended 2 years, people with MDD who had been on a single medication (and not responding) for the last two years would *not* be considered TRD, even if the current episode is longer than 2 years and they had failed other antidepressants before. This might be a problem especially in healthcare systems where PWLE tend to stay for a long period of time on an antidepressant to which they are not responding.

## **Type of medications**

The recommendation of having treatment failures with two medications with different mechanisms could seem rather restrictive compared with other guidelines stating that these two failed treatments could be with any two medications for MDD^10^. As an example, the recent EMA approval of intranasal esketamine in people with TRD considered two treatment failures regardless of the mechanism of action^56^. However, clinically there is some evidence that switching between medications with the same mechanism is less likely to produce a different clinical response, while switching between different classes could be more effective^57^. We debated how accurate is our understanding of the concept of ‘similar vs. different mechanism of action’, even after the NbN has improved our classification. Medications for MDD are pharmacologically complex, and their mechanism of action can be different from the biological (or neurobiological) effects or the downstream physiological effects, and we accept that there is a certain degree of variability in judging whether two different failures are ‘from different classes’ or not. Nevertheless, allowing any combinations of two (failed) medications for MDD regardless of the mechanism of action would allow the inclusion as TRD of individuals that have taken potentially identical drugs, and could (relatively easily) respond to a different drug. Moreover, choosing two drugs from two different classes is consistent with clinical practice^58^.

Some experts supported the inclusion of individuals in whom only one medication for MDD plus an adjunctive agent not licensed for MDD, like aripiprazole or quetiapine, failed to improve or eliminate MDD symptoms. This, not only because they are effective in TRD even in the absence of psychotic features^59^, but also because these drugs have several pharmacological targets and thus could theoretically be considered ‘antidepressants of another class’. However, there are some geographically relevant label limitations to their usage in TRD/PRD. Specifically, in the EU, only extended release (XR) quetiapine is approved for clinical use as add-on treatment in people with MDD who have had sub-optimal response to treatment (thus in PRD or TRD); and, in the US, the olanzapine/fluoxetine combination is approved for TRD, while aripiprazole, brexpiprazole and quetiapine XR are approved as adjunctive treatment for MDD (thus, for people with PRD). Another medication with evidence of effectiveness in augmentation for MDD is lithium^60^ but again this is not FDA or EMA approved in unipolar depression. Based on these considerations, and aware that definitions may diverge from the real-world clinical practice, the consensus was to require that both (failed) treatments should be with two established (licensed) antidepressant agents, again to limit variability within/between different TRD samples, and indeed to make sure that no individual with MDD would be defined TRD after treatment with only one proper antidepressant medication.

Psychotherapy has been proven to be an effective treatment for people with TRD^61^,^62^, but evidence is still sparce and psychological treatments are extremely variable in type and approach. Some experts argued that psychotherapeutic interventions which fail to improve/eliminate MDD symptoms should be regarded as equivalent to pharmacotherapy which fail to improve/eliminate MDD symptoms, if the interventions have been carried out as an evidence-based treatment for depression, such as cognitive behavioural therapy (CBT), while others expressed the opposite opinion, i.e., that the failure to respond to psychotherapy does not imply a greater resistance to pharmacological treatments, although it may indicate a reduced likelihood of placebo response. In our recommendations, we decided *not* to include psychotherapy as one of the two failed treatments. Of note, clinical trials for purposes of regulatory approval, including platform trials, may in the future include trials of psychotherapies or computer-based digital apps as new treatments or as comparisons, and so this recommendation may need to be revisited.

We recommend that the criteria of ‘adequate dose’ is the minimal effective (licensed) dose. Contributors agreed that some individuals only respond to higher doses of an antidepressant, or the maximum licensed dosage, or also the maximum tolerated dosage (which can be even higher than the maximum licensed dosage). For example, venlafaxine is used at higher doses to induce a full noradrenergic effect^63^, and there is some evidence that fluoxetine is more effective at a higher dose than the minimal licensed one^64^. Thus, increasing the dosage can be a reasonable step in the clinical management of some individuals, especially with PRD, and indeed it is an established good clinical practice when a medication is well tolerated and has produced a partial response at a lower dosage. Some experts highlighted the possible consequences of this recommendation; for example, that failure to respond to 50 mg of sertraline would be enough to count as one of the two required treatment failures. However, using higher doses would restrict participants’ selection excessively, to a point where many non-responders would be wrongly excluded from TRD trials because their doses were not pushed up to the maximum tolerated or licenced doses.

## **Exclusion from TRD/PRD studies**

There was debate concerning the maximum number of failed treatments as a potential exclusion criterion, even if the consensus at the end was not to have such criterion. Some experts suggested that people with MTR (failure to 3-5 antidepressants) should not be included in TRD definition, in order to have a more homogeneous group. Indeed, results from the STAR*D study demonstrate that the chances of ameliorating depressive symptoms diminish as additional treatment strategies are used in switching or augmentation because of previous failure^65^. This decrease is particularly relevant after level 2 of the study, in which different treatment options were provided besides the original SSRI, citalopram (given at level 1); this level randomly allowed for a switch (to bupropion, sertraline, or venlafaxine, or cognitive therapy) or augmentation (citalopram plus bupropion, buspirone, or cognitive therapy). After the first two levels, almost half of the individuals achieved remission (37% in level one and 31% in level 2). In level 3, participants were given lithium or a thyroid hormone (triiodothyronine) in augmentation to their reuptake inhibitor, or they were switched to mirtazapine or nortriptyline monotherapy; the proportion of individuals achieving remission dropped to 14%. Finally, level 4 consisted of the non-selective and irreversible MAOI, tranylcypromine, or a combination of venlafaxine and mirtazapine^65^, and at this level, the rate of remission was 13%. This evidence would imply that people who have taken 3 or more ineffective treatment strategies have progressively less chance to respond to switching to/adding a new one, and this consideration would suggest the possibility that such individuals should be excluded from TRD trials. However, notably, low-dose medications for psychosis were not used in STAR*D, which is a widely used strategy nowadays, and a failure to respond to some medications for MDD does not unequivocally imply failure to respond to new treatments. In fact, other contributors to this report argued that novel compounds might work even for individuals with multiple ineffective treatments, and excluding such subjects would be negating their chances of trying new treatments. Other authors^7^ have suggested that a threshold indicating MTR could be used to study these very resistant individuals, or at least to describe how many of them are present in a TRD cohort.

There was some debate on the point of excluding individuals in whom DBS/VNS failed to improve/eliminate MDD symptoms. Some experts argued that there is no evidence to justify the concept that there are some individuals who are so treatment-resistant that they will never respond to anything – not even to medications or interventions that do not currently exist. Some commented that it was arbitrary to include individuals who failed to respond to ECT but not those who failed to respond to VNS/DBS, although others replied that ECT, with its episodic administration and non-invasive approach, would still be offered to individuals that have ‘less severe TRD’ compared with those who are offered VNS/DBS. Overall, we recommend that these individuals should be included in specific research studies and clinical trials for ‘refractory depression’.

## **Clinical presentation**

Some experts raised the issue of potentially excluding a relevant number of PWLE by excluding antecedent and independent personality disorders, which by definition start early in life. On the other hand, others suggested to exclude all the individuals with personality disorders, since these may jeopardise the findings. At the end, we settled for excluding personality disorders (and other psychiatric comorbidities) only if clearly preceding the onset of depression and corroborated, when possible, by health records or other collateral history, and not only by individuals’ accounts and recollections, also because the symptoms overlap between MDD and other psychiatric conditions makes it difficult to distinguish them diagnostically in retrospect^66^. We additionally suggest that individuals with a primary diagnosis of personality disorder or with active and severe substance use (thus, excluded from the present definition) should be included in future research on treatments specifically targeting MDD in those subjects.

We also debated the issue of age. Depression in elderly may be associated with delays in antidepressant response and greater susceptibility to side effects^58^. Moreover, the prevalence of MDD increases in late life, and it is often comorbid with other physical illnesses and consequent polymedication^67^. It is also crucial to distinguish early-onset depression from late-onset depression, as this latter may be subsequent to organic diseases, such as prodromal states of dementia^68^. Because of these differences and complexities, most of the clinical trials for regulatory purposes set a maximum age for inclusion (usually 60-65 years), and we aligned with this view that individuals aged over 65 years should be included in specific clinical trials but not in generic adult TRD ones.

**Diagnostic tools and measures of outcome**

It is important to highlight that currently available outcome measurement tools have some intrinsic problems. Firstly, there is a lack of content overlap between the different tools^69^ for MDD severity. Therefore, by measuring different symptoms, they could potentially provide different classifications of individuals with MDD. In addition, the measurement error at each assessment and the lack of measurement invariance across timepoints may contribute to complicate the identification of a population based on a percentage reduction on clinical scales. Also, while contributors highlighted the importance of tools to aid the systematic collection of data, a raw staging score *per se* (like in the MSM) does not indicate the different elements of the clinical history, like ineffective treatments or illness severity and duration, which should all be separated out when reporting it in order to understand the recruited sample.

Finally, we acknowledge that, especially in research settings, the capacity to measure response occurring within hours rather than days is becoming increasingly relevant, as with esketamine, DBS and psilocybin^70^. Although this report does not recommend the routine use of shorter versions of traditional MDD scales, we acknowledge these may more adequately cover the symptoms of relevance for PWLE in these specific cases.

# **References**

1. Saville BR, Berry SM. Efficiencies of platform clinical trials: A vision of the future. Clinical Trials. 2016.

2. Woodcock J, LaVange LM. Master Protocols to Study Multiple Therapies, Multiple Diseases, or Both. New England Journal of Medicine. 2017.

3. Angus DC, Alexander BM, Berry S, Buxton M, Lewis R, Paoloni M, et al. Adaptive platform trials: definition, design, conduct and reporting considerations. Nature Reviews Drug Discovery. 2019.

4. Cipriani A, Furukawa TA, Salanti G, Chaimani A, Atkinson LZ, Ogawa Y, et al. Comparative efficacy and acceptability of 21 antidepressant drugs for the acute treatment of adults with major depressive disorder: a systematic review and network meta-analysis. The Lancet. 2018.

5. Gaynes BN, Lux L, Gartlehner G, Asher G, Forman‐Hoffman V, Green J, et al. Defining treatment‐resistant depression. Depression and Anxiety. 2019.

6. Salloum NC, Papakostas GI. Staging treatment intensity and defining resistant depression: Historical overview and future directions. Journal of Clinical Psychiatry. 2019.

7. McAllister-Williams RH, Christmas DMB, Cleare AJ, Currie A, Gledhill J, Insole L, et al. Multiple-therapy-resistant major depressive disorder: A clinically important concept. British Journal of Psychiatry. 2018.

8. McAllister-Williams RH, Arango C, Blier P, Demyttenaere K, Falkai P, Gorwood P, et al. The identification, assessment and management of difficult-to-treat depression: An international consensus statement. Journal of Affective Disorders. 2020.

9. Food and Drug Administration. Major Depressive Disorder: Developing Drugs for Treatment, Guidance for Industry’, DRAFT GUIDANCE. U.S. Department of Health and Human Services, Food and Drug Administration, Center for Drug Evaluation and Research (CDER), Revision 1. 2018.

10. European Medicines Agency. Guideline on clinical investigation of medicinal products in the treatment of depression. EMA/CHMP/185423/2010 Rev 2. 2013.

11. Jackson WC, Papakostas GI, Rafeyan R, Trivedi MH. Recognizing Inadequate Response in Patients With Major Depressive Disorder. The Journal of clinical psychiatry. 2020.

12. First, M. B., Spitzer, R.L, Gibbon M., and Williams JBW. Structured Clinical Interview for DSM-IV-TR Axis I Disorders. New York State Psychiatric Institute. 2002.

13. First MB, Williams JBW, Karg RS, Spitzer RL. Structured clinical interview for DSM-5 research version. American Psychiatric Association, Washington DC. 2015.

14. Sheehan D V., Lecrubier Y, Sheehan KH, Amorim P, Janavs J, Weiller E, et al. The Mini-International Neuropsychiatric Interview (M.I.N.I.): The development and validation of a structured diagnostic psychiatric interview for DSM-IV and ICD-10. In: Journal of Clinical Psychiatry. 1998.

15. Chandler GM, Iosifescu D V., Pollack MH, Targum SD, Fava M. Validation of the massachusetts general hospital Antidepressant Treatment History Questionnaire (ATRQ). CNS Neuroscience and Therapeutics. 2010.

16. Sackeim HA. The definition and meaning of treatment-resistant depression. Journal of Clinical Psychiatry. 2001.

17. Desseilles M, Witte J, Chang TE, Iovieno N, Dording CM, Ashih H, et al. Assessing the adequacy of past antidepressant trials: A clinician’s guide to the antidepressant treatment response questionnaire. Journal of Clinical Psychiatry. 2011.

18. Sackeim HA, Aaronson ST, Bunker MT, Conway CR, Demitrack MA, George MS, et al. The assessment of resistance to antidepressant treatment: Rationale for the Antidepressant Treatment History Form: Short Form (ATHF-SF). Journal of Psychiatric Research. 2019.

19. Ruhé HG, Van Rooijen G, Spijker J, Peeters FPML, Schene AH. Staging methods for treatment resistant depression. A systematic review. Journal of Affective Disorders. 2012.

20. Thase ME, Rush AJ. When at first you don’t succeed: Sequential strategies for antidepressant nonresponders. In: Journal of Clinical Psychiatry. 1997.

21. Thase ME, John Rush A, Howland RH, Kornstein SG, Kocsis JH, Gelenberg AJ, et al. Double-blind switch study of imipramine or sertraline treatment of antidepressant-resistant chronic depression. Archives of General Psychiatry. 2002.

22. Köhler-Forsberg O, Larsen ER, Buttenschon HN, Rietschel M, Hauser J, Souery D, et al. Effect of antidepressant switching between nortriptyline and escitalopram after a failed first antidepressant treatment among patients with major depressive disorder. British Journal of Psychiatry. 2019.

23. Fava M. Diagnosis and definition of treatment-resistant depression. Biological Psychiatry. 2003.

24. Fekadu A, Wooderson S, Donaldson C, Markopoulou K, Masterson B, Poon L, et al. A multidimensional tool to quantify treatment resistance in depression: The Maudsley staging method. Journal of Clinical Psychiatry. 2009.

25. Van Belkum SM, Geugies H, Lysen TS, Cleare AJ, Peeters FPML, Penninx BWJH, et al. Validity of the maudsley staging method in predicting treatment-resistant depression outcome using the netherlands study of depression and anxiety. Journal of Clinical Psychiatry. 2018.

26. Fekadu A, Donocik JG, Cleare AJ. Standardisation framework for the Maudsley staging method for treatment resistance in depression. BMC Psychiatry. 2018.

27. Conway CR, George MS, Sackeim HA. Toward an evidence-based, operational definition of treatment-resistant depression: When Enough is enough. Vol. 74, JAMA Psychiatry. American Medical Association; 2017. p. 9–10.

28. Felger JC, Li Z, Haroon E, Woolwine BJ, Jung MY, Hu X, et al. Inflammation is associated with decreased functional connectivity within corticostriatal reward circuitry in depression. Molecular Psychiatry. 2016.

29. Williams B, Mancia G, Spiering W, Rosei EA, Azizi M, Burnier M, et al. 2018 ESC/ESH Guidelines for themanagement of arterial hypertension. European Heart Journal. 2018.

30. Fabbri C, Hagenaars SP, John C, Williams AT, Shrine N, Moles L, et al. Genetic and clinical characteristics of treatment-resistant depression using primary care records in two UK cohorts. Molecular Psychiatry 2021. 2021 Mar 22;1–11.

31. Kirchheiner J, Meineke I, Müller G, Bauer S, Rohde W, Meisel C, et al. Influence of CYP2C9 and CYP2D6 Polymorphisms on the Pharmacokinetics of Nateglinide in Genotyped Healthy Volunteers. Clinical Pharmacokinetics 2004 43:4. 2012 Sep 30;43(4):267–78.

32. Cattaneo A, Ferrari C, Turner L, Mariani N, Enache D, Hastings C, et al. Whole-blood expression of inflammasome- and glucocorticoid-related mRNAs correctly separates treatment-resistant depressed patients from drug-free and responsive patients in the BIODEP study. Translational Psychiatry. 2020.

33. Wittenberg GM, Greene J, Vértes PE, Drevets WC, Bullmore ET. Major Depressive Disorder Is Associated With Differential Expression of Innate Immune and Neutrophil-Related Gene Networks in Peripheral Blood: A Quantitative Review of Whole-Genome Transcriptional Data From Case-Control Studies. Biological Psychiatry. 2020.

34. Lynall ME, Turner L, Bhatti J, Cavanagh J, de Boer P, Mondelli V, et al. Peripheral Blood Cell–Stratified Subgroups of Inflamed Depression. Biological Psychiatry. 2020.

35. Uher R, Tansey KE, Dew T, Maier W, Mors O, Hauser J, et al. An inflammatory biomarker as a differential predictor of outcome of depression treatment with escitalopram and nortriptyline. American Journal of Psychiatry. 2014.

36. Chamberlain SR, Cavanagh J, De Boer P, Mondelli V, Jones DNC, Drevets WC, et al. Treatment-resistant depression and peripheral C-reactive protein. British Journal of Psychiatry. 2019.

37. Nouraei H, Firouzabadi N, Mandegary A, Zomorrodian K, Bahramali E, Shayesteh MRH, et al. Glucocorticoid receptor genetic variants and response to fluoxetine in major depressive disorder. Journal of Neuropsychiatry and Clinical Neurosciences. 2018.

38. O’Connell CP, Goldstein-Piekarski AN, Nemeroff CB, Schatzberg AF, Debattista C, Carrillo-Roa T, et al. Antidepressant outcomes predicted by Genetic variation in corticotropin-releasing hormone binding protein. American Journal of Psychiatry. 2018.

39. Uhr M, Tontsch A, Namendorf C, Ripke S, Lucae S, Ising M, et al. Polymorphisms in the Drug Transporter Gene ABCB1 Predict Antidepressant Treatment Response in Depression. Neuron. 2008.

40. Porcelli S, Fabbri C, Serretti A. Meta-analysis of serotonin transporter gene promoter polymorphism (5-HTTLPR) association with antidepressant efficacy. European Neuropsychopharmacology. 2012.

41. Murphy GM, Kremer C, Rodrigues HE, Schatzberg AF. Pharmacogenetics of antidepressant medication intolerance. American Journal of Psychiatry. 2003.

42. Anttila S, Huuhka K, Huuhka M, Rontu R, Hurme M, Leinonen E, et al. Interaction between 5-HT1A and BDNF genotypes increases the risk of treatment-resistant depression. Journal of Neural Transmission. 2007.

43. Akil H, Gordon J, Hen R, Javitch J, Mayberg H, McEwen B, et al. Treatment resistant depression: A multi-scale, systems biology approach. Neuroscience and Biobehavioral Reviews. 2018.

44. Krystal AD, Pizzagalli DA, Mathew SJ, Sanacora G, Keefe R, Song A, et al. The first implementation of the NIMH FAST-FAIL approach to psychiatric drug development. Nature Reviews Drug Discovery. 2018.

45. Chevance A, Ravaud P, Tomlinson A, Le Berre C, Teufer B, Touboul S, et al. Identifying outcomes for depression that matter to patients, informal caregivers, and health-care professionals: qualitative content analysis of a large international online survey. The Lancet Psychiatry. 2020.

46. Mäntylä FL. Major Depressive Disorder - A Patient Perspective to Recovery. Inspire The Mind. 2020.

47. Laursen DRT, Paludan-Müller AS, Hróbjartsson A. Randomized clinical trials with run-in periodsfrequency, characteristics and reporting. Clinical Epidemiology. 2019.

48. Kennedy SH, Lam RW, McIntyre RS, Tourjman SV, Bhat V, Blier P, et al. Canadian Network for Mood and Anxiety Treatments (CANMAT) 2016 clinical guidelines for the management of adults with major depressive disorder: Section 3. Pharmacological Treatments. Canadian Journal of Psychiatry. 2016.

49. Thase ME, Trivedi MH, Nelson JC, Fava M, Swanink R, Tran Q Van, et al. Examining the efficacy of adjunctive aripiprazole in major depressive disorder: A pooled analysis of 2 studies. Primary Care Companion to the Journal of Clinical Psychiatry. 2008.

50. Daly EJ, Singh JB, Fedgchin M, Cooper K, Lim P, Shelton RC, et al. Efficacy and safety of intranasal esketamine adjunctive to oral antidepressant therapy in treatment-resistant depression: A randomized clinical trial. JAMA Psychiatry. 2018.

51. McIntyre RS, Rosenblat JD, Nemeroff CB, Sanacora G, Murrough JW, Berk M, et al. Synthesizing the Evidence for Ketamine and Esketamine in Treatment-Resistant Depression: An International Expert Opinion on the Available Evidence and Implementation. 2021 Mar 17;178(5):383–99.

52. Buckman JEJ, Underwood A, Clarke K, Saunders R, Hollon SD, Fearon P, et al. Risk factors for relapse and recurrence of depression in adults and how they operate: A four-phase systematic review and meta-synthesis. Clinical Psychology Review. 2018.

53. Fava GA, Offidani E. The mechanisms of tolerance in antidepressant action. Progress in Neuro-Psychopharmacology and Biological Psychiatry. 2011.

54. Voineskos D, Daskalakis ZJ, Blumberger DM. Management of treatment-resistant depression: Challenges and strategies. Neuropsychiatric Disease and Treatment. 2020.

55. Luo Y, Kataoka Y, Ostinelli EG, Cipriani A, Furukawa TA. National Prescription Patterns of Antidepressants in the Treatment of Adults With Major Depression in the US Between 1996 and 2015: A Population Representative Survey Based Analysis. Frontiers in Psychiatry. 2020.

56. European Medicines Agency. Spravato (esketamine) An overview of Spravato and why it is authorised in the EU. EMA/578240/2019 EMEA/H/C/004535; 2019.

57. Papakostas GI, Fava M, Thase ME. Treatment of SSRI-Resistant Depression: A Meta-Analysis Comparing Within- Versus Across-Class Switches. Biological Psychiatry. 2008.

58. Taylor, D.M., Barnes, T.R. and Young AH. The Maudsley prescribing guidelines in psychiatry. John Wiley & Sons.; 2018.

59. Nelson JC, Papakostas GI. Atypical antipsychotic augmentation in major depressive disorder: A meta-analysis of placebo-controlled randomized trials. American Journal of Psychiatry. 2009.

60. Bauer M, Adli M, Ricken R, Severus E, Pilhatsch M. Role of lithium augmentation in the management of major depressive disorder. CNS Drugs. 2014.

61. Van Bronswijk S, Moopen N, Beijers L, Ruhe HG, Peeters F. Effectiveness of psychotherapy for treatment-resistant depression: A meta-analysis and meta-regression. Psychological Medicine. 2019.

62. Gloster AT, Rinner MTB, Ioannou M, Villanueva J, Block VJ, Ferrari G, et al. Treating treatment non-responders: A meta-analysis of randomized controlled psychotherapy trials. Clinical Psychology Review. 2020.

63. Debonnel G, Saint-André É, Hébert C, De Montigny C, Lavoie N, Blier P. Differential physiological effects of a low dose and high doses of venlafaxine in major depression. International Journal of Neuropsychopharmacology. 2007.

64. Furukawa TA, Cipriani A, Cowen PJ, Leucht S, Egger M, Salanti G. Optimal dose of selective serotonin reuptake inhibitors, venlafaxine, and mirtazapine in major depression: a systematic review and dose-response meta-analysis. The Lancet Psychiatry. 2019.

65. Rush AJ, Trivedi MH, Wisniewski SR, Nierenberg AA, Stewart JW, Warden D, et al. Acute and longer-term outcomes in depressed outpatients requiring one or several treatment steps: A STAR*D report. American Journal of Psychiatry. 2006.

66. Regier DA, Narrow WE, Clarke DE, Kraemer HC, Kuramoto SJ, Kuhl EA, et al. DSM-5 field trials in the United States and Canada, part II: Test-retest reliability of selected categorical diagnoses. American Journal of Psychiatry. 2013.

67. Cheruvu VK, Chiyaka ET. Prevalence of depressive symptoms among older adults who reported medical cost as a barrier to seeking health care: findings from a nationally representative sample. BMC geriatrics. 2019.

68. Yalin N, Young AH. The age of onset of unipolar depression. In: Age of Onset of Mental Disorders: Etiopathogenetic and Treatment Implications. 2018.

69. Fried EI. The 52 symptoms of major depression: Lack of content overlap among seven common depression scales. Journal of Affective Disorders. 2017.

70. Carhart-Harris RL, Bolstridge M, Rucker J, Day CMJ, Erritzoe D, Kaelen M, et al. Psilocybin with psychological support for treatment-resistant depression: an open-label feasibility study. The Lancet Psychiatry. 2016.

# ***Table S1:*** Table of contributors

| **Contributors** | | |
| --- | --- | --- |
| **Name** | | **Area of expertise** |
| ***Academic and clinical experts*** | | |
| ***1*** | Ian M. Anderson | *Clinical psychopharmachology* |
| ***2*** | Bruno Aouizerate | *Clinical psychopharmacology and neuromodulation* |
| ***3*** | Volker Arolt | *Basic and clinical psychopharmacology, psychotherapy and neuromodulation* |
| ***4*** | Gara Arteaga-Henríquez | *Basic and clinical psychopharmacology and neuromodulation* |
| ***5*** | Michael Bauer | *Clinical psychopharmacology, clinical trials* |
| ***6*** | Bernhard T. Baune | *Clinical psychopharmacology, psychotherapy and neuromodulation* |
| ***7*** | Francesco Benedetti | *Basic and clinical psychopharmacology and neuromodulation* |
| ***8*** | Pierre Blier | *Basic and clinical psychopharmacology, psychotherapy and neuromodulation* |
| ***9*** | Lucinda Cash-Gibson | *Clinical trials and public health* |
| ***10*** | Woo-Ri Chae | *Basic and clinical psychopharmacology and epidemiology* |
| ***11*** | Anthony J. Cleare | *Basic and clinical psychopharmacology* |
| ***12*** | Philip J. Cowen | *Basic and clinical psychopharmacology* |
| ***13*** | Timothy G. Dinan | *Basic and clinical psychopharmacology* |
| ***14*** | Andrea Fagiolini | *Clinical psychopharmacology* |
| ***15*** | I. Nicol Ferrier | *Clinical psychopharmacology and neuromodulation* |
| ***16*** | Stefan M. Gold | *Basic and clinical psychopharmacology* |
| ***17*** | Ulrich Hegerl | *Clinical psychopharmacology, psychotherapy and neuromodulation* |
| ***18*** | Witte J. G. Hoogendijk | *Basic and clinical psychopharmacology and psychotherapy* |
| ***19*** | Melisa Kose | *Basic psychopharmacology and clinical trials* |
| ***20*** | Andrew D. Krystal | *Clinical psychopharmacology and neuromodulation* |
| ***21*** | Marion Leboyer | *Clinical psychopharmacology and neuromodulation* |
| ***22*** | Eduard Maron | *Basic and clinical psychopharmacology, digital tools* |
| ***23*** | R. Hamish McAllister-Williams | *Clinical psychopharmacology and neuromodulation* |
| ***24*** | Roger S. McIntyre | *Clinical psychopharmacology* |
| ***25*** | Elisa Melloni | *Clinical psychology, psychotherapy and neuromodulation* |
| ***26*** | Andreas Meyer-Lindenberg | *Basic and clinical psychopharmacology* |
| ***27*** | Andrew H. Miller | *Basic and clinical psychopharmacology* |
| ***28*** | Charles B. Nemeroff | *Basic and clinical psychopharmacology, psychotherapy and neuromodulation* |
| ***29*** | Claus Normann | *Basic and clinical psychopharmacology, psychotherapy and neuromodulation* |
| ***30*** | David Nutt | *Clinical psychopharmacology and psychotherapy* |
| ***31*** | Christian Otte | *Clinical psychopharmacology and psychotherapy* |
| ***32*** | Stefano Pallanti | *Clinical psychopharmacology and neuromodulation* |
| ***33*** | Luca Pani | *Basic and clinical psychopharmacology, regulatory science* |
| ***34*** | Carmine M. Pariante | *Basic and clinical psychopharmacology* |
| ***35*** | Brenda W. J. H. Penninx | *Clinical psychopharmacology* |
| ***36*** | Gabriela Perez-Fuentes | *Clinical psychology and psychotherapy* |
| ***37*** | Sara Poletti | *Basic and clinical psychopharmacology and neuromodulation* |
| ***38*** | J. Antoni Ramos-Quiroga | *Basic and clinical psychopharmacology* |
| ***39*** | Alan F. Schatzberg | *Clinical psychopharmacology, psychotherapy and neuromodulation* |
| ***40*** | Koen Schruers | *Clinical psychopharmacology, psychotherapy, neuromodulation and regulatory science* |
| ***41*** | Luca Sforzini | *Basic and clinical psychopharmacology* |
| ***42*** | Richard C. Shelton | *Clinical psychopharmacology and psychotherapy* |
| ***43*** | Edwin van de Ketterij | *Basic psychopharmacology and clinical trials* |
| ***44*** | Courtney Worrell | *Clinical trials* |
| ***45*** | Lakshmi N. Yatham | *Clinical psychopharmacology, psychotherapy and neuromodulation* |
| ***46*** | Allan H. Young | *Basic and clinical psychopharmacology and neuromodulation* |
| ***47*** | Roland Zahn | *Clinical psychopharmacology, psychotherapy and neuromodulation* |
| ***Representatives of regulatory authorities*** | | |
| ***48*** | Georgios Aislaitner | *Regulatory science* |
| ***49*** | Florence Butlen-Ducuing | *Regulatory science, clinical psychopharmacology* |
| ***50*** | Marion Haberkamp | *Regulatory science* |
| ***51*** | Thomas Laughren | *Clinical trials and regulatory science* |
| ***52*** | Andrew Thomson | *Regulatory science* |
| ***People with lived experience (PWLE)*** | | |
| ***53*** | Fanni-Laura Mäntylä | *PWLE* |
| ***Employees of pharmaceutical companies*** | | |
| ***54*** | Heidi De Smedt | *Industry and clinical trials* |
| ***55*** | Christine Fletcher | *Clinical trials and regulatory science* |
| ***56*** | Yanina Flossbach | *Clinical psychopharmacology, industry, psychotherapy and neuromodulation* |
| ***57*** | Valeria Jordán Mondragón | *Clinical psychopharmacology, industry and psychotherapy* |
| ***58*** | Jadwiga Martynowicz | *Industry and regulatory science* |
| ***59*** | Adam J. Savitz | *Clinical psychopharmacology, industry, regulatory science and clinical trials* |
| ***60*** | Mark E. Schmidt | *Industry and clinical trials* |
| ***61*** | Katherine Woo | *Industry and clinical trials* |

# ***Table S2:*** CREDES checklist of the Delphi process

| **Items of reporting** | **Reported on page** |
| --- | --- |
| *Purpose and rationale.* The purpose of the study should be clearly defined and demonstrate the appropriateness of the use of the Delphi technique as a method to achieve the research aim. A rationale for the choice of the Delphi technique as the most suitable method needs to be provided. | -Main Document, page 7 |
| *Expert panel.* Criteria for the selection of experts and transparent information on recruitment of the expert panel, sociodemographic details including information on expertise regarding the topic in question, (non)response and response rates over the ongoing iterations should be reported. | -Main Document, pages 8-9  -Appendix, section *“Delphi process for this report”* pages 4-5  -Appendix, Table S1 |
| *Description of the methods.* The methods employed need to be comprehensible; this includes information on preparatory steps (How was available evidence on the topic in question synthesised?), piloting of material and survey instruments, design of the survey instrument(s), the number and design of survey rounds, methods of data analysis, processing and synthesis of experts’ responses to inform the subsequent survey round and methodological decisions taken by the research team throughout the process. | -Main Document,  section *“Our Delphi-method-based approach”*, pages 8-9  -Appendix, sections *“Delphi process for this report”*, pages 4-5 and *“Review of the literature”*, pages 5-7  -Main Document, Figure 1 |
| *Procedure.* Flow chart to illustrate the stages of the Delphi process, including a preparatory phase, the actual ‘Delphi rounds’, interim steps of data processing and analysis, and concluding steps. | -Main Document, Figure 1 |
| *Definition and attainment of consensus.* It needs to be comprehensible to the reader how consensus was achieved throughout the process, including strategies to deal with non-consensus. | -Main Document, page 9  -Main Document, Table 1  -Main Document, Figure 2 |
| *Results*. Reporting of results for each round separately is highly advisable in order to make the evolving of consensus over the rounds transparent. This includes figures showing the average group response, changes between rounds, as well as any modifications of the survey instrument such as deletion, addition or modification of survey items based on previous rounds. | -Main Document, Table 1  -Main Document, Figure 1  -Appendix, Supplementary file S3 |
| *Discussion of limitations.* Reporting should include a critical reflection of potential limitations and their impact of the resulting guidance. | -Main Document, section *“Limitations and conclusions”*, pages 24-25 |
| *Adequacy of conclusions.* The conclusions should adequately reflect the outcomes of the Delphi study with a view to the scope and applicability of the resulting practice guidance. | -Main Document, page 25 |
| *Publication and dissemination*. The resulting guidance on good practice in palliative care should be clearly identifiable from the publication, including recommendations for transfer into practice and implementation. If the publication does not allow for a detailed presentation of either the resulting practice guidance or the methodological features of the applied Delphi technique, or both, reference to a more detailed presentation elsewhere should be made (e.g. availability of the full guideline from the authors or online; publication of a separate paper reporting on methodological details and particularities of the process (e.g. persistent disagreement and controversy on certain issues)). A dissemination plan should include endorsement of the guidance by professional associations and health care authorities to facilitate implementation. | -Main Document, page 9  -Main Document, Table 1  -Main Document, Figure 2 |
| *Jünger S, Payne SA, Brine J, Radbruch L, Brearley SG. Guidance on Conducting and REporting DElphi Studies (CREDES) in palliative care: Recommendations based on a methodological systematic review. Palliat Med. 2017;31: 684–706. doi:10.1177/0269216317690685.* | |

# ***Figure S1:*** Systematic review flow diagram

***Identification***

- MEDLINE PubMed® database

*Search string: ((Treatment-resistant depression) or (TRD) or (partially responsive depression) or (PRD) or (difficult-to-treat depression) or (DTD)) and ((definition) or (diagnosis) or (criteria))*

- Reviews, systematic reviews, meta-analyses, and guidelines
- Published from the 1^st^ of March 2020 to the 22^nd^ of January 2021

50 records identified

***Screening and eligibility***  *Excluded*

30 articles not assessing individuals with non-responsive MDD

18 articles not providing rationale for TRD/PRD definitions

***Inclusion***

**2 studies included in systematic review**

# ***Supplementary File S1:*** 1^st^ meeting agenda

EU-PEARL WP4

**Consensus Meeting**

*22^nd^ May 2020*

*Morning session:* 09^00^-12^00^ BST / 04^00^-07^00^ EDT / 01^00^-04^00^ PDT

*Afternoon session:* 15^00^-18^00^ BST / 10^00^-13^00^ EDT / 07^00^-10^00^ PDT

**Treatment-response in Major Depressive Disorder**

**Participants**

| *International experts:* | |  |  |  |
| --- | --- | --- | --- | --- |
| *Morning session* | Ian Anderson |  | *Afternoon session* | Pierre Blier |
|  | Bruno Aouizerate |  |  | Ulrich Hegerl |
|  | Volker Arolt |  |  | Andrew D. Krystal |
|  | Bernhard Baune |  |  | Andrew H. Miller |
|  | Michael Bauer |  |  | Charles B. Nemeroff |
|  | Anthony Cleare |  |  | David Nutt |
|  | Philip Cowen |  |  | Claus Normann |
|  | Ted Dinan |  |  | Stefano Pallanti |
|  | Andrea Fagiolini |  |  | Luca Pani |
|  | Nicol Ferrier |  |  | Roger McIntyre |
|  | Marion Leboyer |  |  | Allan F. Schatzberg |
|  | Hamish McAllister-Williams | |  | Richard C. Shelton |
|  | Andreas Meyer-Lindenberg | |  | Lakshmi Yatham |
|  | Brenda Penninx | |  | Roland Zahn |
|  | Allan Young | |  |  |

*EU-PEARL WP4 leaders:*

Yanina Flossbach

Stefan Gold

Eduard Maron

Christian Otte

Carmine M. Pariante

Josep Antoni Ramos-Quiroga

Adam Savitz

*EU-PEARL WP4 contributors:*

Francesco Benedetti

Gara Arteaga Henriquez

Witte Hoogendijk

Heddie Martynowicz

Luca Sforzini

Courtney Worrell

| Schedule (BST) | Session | Chair | Speakers |
| --- | --- | --- | --- |
| 9^00^-9^15^  15^00^-15^15^ | Introduction |  | Carmine M. Pariante |
| 9^15^-9^25^  15^15^-15^25^ | 1^st^ topic - *Is it helpful to separate TRD vs PRD or is it all a continuous spectrum?* | Carmine M. Pariante | Bernhard Baune & Ted Dinan  Charles B. Nemeroff & Richard C. Shelton |
| 9^25^-9^45^  15^25^-15^45^ | 1^st^ topic discussion |  | Whole group |
| 9^45^-9^55^  15^45^-15^55^ | 2^nd^ topic – *Can we really assess treatment failure historically, beyond the current episode?* | Christian Otte  Stefan M. Gold | Nicol Ferrier & Brenda Penninx  Roger McIntyre & Luca Pani |
| 9^55^-10^15^  15^55^-16^15^ | 2^nd^ topic discussion |  | Whole group |
| 10^15^-10^30^  16^15^-16^30^ | Future research - *Clinical practice vs. Regulatory needs* | Carmine M. Pariante | Ian Anderson  Lakshmi Yatham |
| 10^30^-10^45^  16^30^-16^45^ | Break |  |  |
| 10^45^-10^55^  16^45^-16^55^ | 3^rd^ topic – *Is 4 weeks of AD at the minimal therapeutic dose enough to demonstrate treatment resistance?* | Josep Antoni Ramos-Quiroga  Adam Savitz | Philip Cowen & Michael Bauer  Ulrich Hegerl & David Nutt |
| 10^55^-11^15^  16^55^-17^15^ | 3^rd^ topic discussion |  | Whole group |
| 11^15^-11^25^  17^15^-17^25^ | 4^th^ topic - *Is TRD comorbid with personality disorder or substance abuse a different psychiatric disorder?* | Yanina Flossbach  Eduard Maron | Allan Young & Anthony Cleare  Pierre Blier & Alan F. Schatzberg |
| 11^25^-11^45^  17^25^-17^45^ | 4^th^ topic discussion |  | Whole group |
| 11^45^-12^00^  17^45^-18^00^ | Future research – *Cluster of symptoms and biomarkers* | Carmine M. Pariante | Andrea Fagiolini  Andrew H. Miller |

# ***Supplementary File S2:*** 2^nd^ meeting agenda

EU-PEARL WP4

**Stakeholder Meeting**

*9^th^ October 2020*

15^00^-17^00^ BST (London time) / 10^00^-12^00^ EDT (New York time)

**Treatment-response in Major Depressive Disorder**

| Schedule (BST) | Session | Speakers / Chair |
| --- | --- | --- |
| 15^00^-15^10^ | Introduction | Carmine M. Pariante |
| 15^10^-15^30^ | *EU-PEARL project and main objectives in MDD* | Luca Sforzini & Courtney Worrell |
| 15^35^-15^50^ | *Operational criteria for TRD and PRD definitions (1^st^)* | Luca Sforzini |
| 15^50^-16^15^ | Discussion – *Whole group* | Adam Savitz & Yanina Flossbach |
| 16^15^-16^30^ | *Operational criteria for TRD and PRD definitions (2^nd^)* | Luca Sforzini |
| 16^30^-16^55^ | Discussion – *Whole group* | Stefan Gold & Jadwiga Martynowicz |
| 16^55^-17^00^ | Concluding remarks | Carmine M. Pariante |

# ***Supplementary File S3:*** Introductory report

**EU-PEARL WP4**

**Introductory report**

*(For the Consensus Meeting)*

**1) Do you think that it is useful to have a definition of TRD for clinical trials conducted for regulatory purposes?**

We have an almost total consensus on this question. Twenty-three experts agreed on the necessity to have a clear definition of TRD for clinical trials conducted for regulatory purposes, and many of them were extremely convinced about it. However, some have argued that this definition could be meaningless in a clinical perspective, and that this could be arbitrary and influenced by different healthcare systems. Moreover, it might be worth checking new compounds in general MDD not only in TRD. As mentioned above, the report is specifically dedicated to defining the populations for regulatory clinical trials, but we will now discuss this issue in a dedicated session.

**2a) Do you think that it is useful to differentiate between TRD and PRD?**

The majority of the replies were “yes” (n=16). However, there has been debate around this concept. Someone replied it is absolutely useful to differentiate between TRD and PRD because this issue cuts at the heart of the problem, or that this is even mandatory, because TRD and PRD are two clinically different conditions that lead to different responses, or because treatment strategies will be very different and conceptually, they may be different “organisms”. Conversely, some other experts seem more sceptical about this differentiation. For example, a point was raised that even though PRD is a real thing, this differentiation could be more of a problem, being not really translatable into regulatory definitions. Other experts were even more openly sided with the “no”, saying for example that the problem with TRD is that no new novel drug therapies have emerged, and semantic discussion on terminology is not going to help. Others argued that this differentiation is not recommended, since TRD is a continuum and, in addition, a lack of partial response may not make a patient more resistant to a new strategy. An interesting alternative which come out from the comments would be to use a dimensional approach rather than a categorical one. This important issue will be discussed in *Session 1*.

**2b) If so, should it be based only on history (response to previous antidepressants) or also on current depressive symptoms while on antidepressants?**

Here, even though there is a consensus on the importance of current depressive symptoms, the opinions are more split. The two main currents of thought are those who think it would be better to include both previous and current episodes in the definition, and those who would rather prefer to consider only the current episode. Indeed, some argue that is equally important to consider also the history of the disease and past episodes besides current symptoms, while others are more convinced that the evaluation should be based on current episode only, mainly because of the unreliability of retrospective gathering of prior treatment responses. This important issue will be discussed *in Session 2*.

**2c) How can patients’ preferences and attitudes be included in this definition?**

For this question there was a substantial agreement on the value of including patients’ preferences in the definition, which could bring us “a movement away from one size fits all”. Different options have been proposed, mainly with a precise focus on which symptoms are the most disabling for the patient and also on functional outcomes, which may be more important than purely symptomatic ones. Some experts suggest here the use of specific scales, such as Quality of Life (QoL) scales, specific checklists, or Visual Analogue Scale (VAS). As mentioned above, this discussion is relevant for the development of future protocols to assess depressed patients longitudinally or to improve the future clinical trial for TRD/PRD, both of which are key aims of this IMI European Union research programme. We will capture the different points of view in the document.

**3a) How many failed prior treatment trials do you think are necessary to define PRD-TRD?**

Consistently with the most commonly used criteria, most of the experts agree on the fact that TRD should be defined after a minimum of two failed trials (n=21). However, some reason that probably a defined number is not needed since we are talking about a continuum from treatment naïve to ultra-TRD, so probably a dimensional system could be better. Moreover, there is great difficulty in defining an episode, and there is a big difference whether the episode started few months or few years before the assessment. So, it could be probably better if a defined period would be set (for example, within the last two years). Some of this debate will be captured in *Session 1*, especially for the ‘continuum’ argument.

**3b) Should the number of failed trials include the current episode?**

Nearly everyone agrees on this point. The main discussion was, once again, if to include the current episode only, and therefore this will be part of *Session 2*.

**3c) If it is two or more, should at least one antidepressant trial be done prospectively as part of a regulatory clinical trial, before testing the novel compound?**

This was a debated issue. Some of the expert think that at least one antidepressant trial should be done prospectively, because of the intrinsic greater reliability of controlled conditions. However, for many experts, this is useful but not essential. In fact, it would be too restrictive in an under-researched area, and risking to reduce the number of eligible patients and select only severely ill patients. In addition, there is an overall concern, considering that the aim of this report is to support future regulatory clinical trials for new medications, that such recommendation would make trials too difficult and expensive. Interestingly, an important suggestion was to use a pharmacological record from the pharmacy or the hospital record to assess the degree of compliance in the current episode, thus making retrospective assessment more reliable. This will be partly discussed in *Session 2*.

**3d) Should a maximum number of failed previous treatments for patients be included in regulatory trials, to select a group which is still amenable to some improvement?**

The most frequent answers to this question were either “no maximum number” or “it depends on clinical scenario”. Both these answers describe the complexity of this topic. The main suggestion seems to be that we should not exclude multiple-resistant patients, but rather include staging to allow good description of the population. Indeed, the number of failed trials could be used as a moderator in the analysis, since it is possible that some novel compounds might work even for these patients with severe TRD, and by excluding such subjects we are negating the chances of finding new treatments for such patients. As some of the experts suggested, different interventions are per se index of more severe resistance; examples are lithium augmentation, ECT treatment, or DBS. The type of intervention should obviously be considered in the number of previous treatments for patients to be included in regulatory trials (*as discussed in the question 11*). On the other hand, we still have several experts who are in favour of defining a maximum number of previous treatments, mostly from three to five. Interestingly, defining a specific number of treatments here could be more useful for research purposes rather than for regulatory purposes. We have not been able to include specific discussion of this topic, so we will use the report to express a consensus.

**4a) Do you think that some types of symptoms should either be prerequisite, or should be excluded, from the definition of TRD? For example, should the presence of melancholic, atypical or anxious symptoms be only recorded, or should it be used to guide inclusion or exclusion?**

Regarding this point, there is a very good consensus that we should consider all the symptoms in the definition, as these are a significant part of the clinical picture. Indeed, the majority suggested to adequately document and record these symptoms, particularly atypical and psychotic symptoms. Results on these clusters could then encourage specific research in this area, promoting specific drug licences and clinical trials using these as inclusion criteria. Within this question there was also a larger debate on the need to increase the knowledge regarding specific pathologies that underpin specific symptoms and may have specific druggable targets. The issue is whether, in the larger framework of TRD, there are specific symptom profiles that might be targeted by novel treatments, such as anhedonia, cognitive symptoms, and vegetative symptoms. In the future, it would probably make sense not to treat all TRD patients the same, and identify subgroups for targeting of persisting symptoms. This is an important theoretical issue which will be discussed in the *Future research* *Session*.

**4b) How about comorbidity with personality disorders or substance abuse?**

Here, even though the majority took position against the inclusion of personality disorder and substance abuse, there was some disagreement. Some experts indeed suggested that one or both of these conditions should be included, and then of course treatment response assessed in each of the subtypes. Because substance abuse and personality disorders are so frequently co-morbid that cannot easily be excluded, some have argued that they have to be included unless they are an active substance use disorder or a primary diagnosis of personality disorder with onset documented by age 18-21. The issue of comorbidity is essential, and for this reason we have dedicated *Session 4* to this topic, with the provocative title: “*Is TRD comorbid with personality disorder or substance abuse a different psychiatric disorder?*”

**5) Are there any genetic markers that you would suggest in the identification of TRD patients, or to further stratify them? Are these tests ready to be used currently (sufficient validity, sensitivity, and specificity)?**

Again, also for this question there was a general consensus that no, there are no genetic/biological markers ready to use for inclusion/exclusion in clinical trials. However, there were some suggestions for markers that were either promising options or, for some experts, currently ready to use. Amongst the most reported we find cytochrome P450 polymorphisms, especially CYP2D6 and CYP2C19, but also other markers, such as genes related to immune function, HPA axis, serotonin transporter, and olfactomedin-4, as well as levels of inflammation (for example, CRP) to predict lack of response in general or to SSRIs in particular. Again, this topic will be briefly discussed in the *Future research Session*.

**6a) Which dosage should be considered when defining a treatment failure, the minimal approved dosage or the maximum approved dosage? Or something else?**

Here, we have many different opinions. One position is that we should go for the maximum licensed dosage, or the maximum tolerated dosage, which could be also beyond recommended dosage. The other strong position is the minimal effective dosage, because the evidence for higher doses, especially for SSRIs, is little and too variable. This issue will be discussed in *Session 3*, together with the minimal duration of treatment.

**6b) How do we assess compliance? Should medications level be conducted at least to confirm current treatment failure?**

Most of the opinions here converge on the utility of performing drug blood levels tests, mainly in experimental trials, given that this is probably too difficult to do for clinical implementation at the moment. Others, however, believe there is no need to perform these tests because the evidence is poor and there is too much variance due to unknown individual factors. Some experts suggested the potential use of specific methods to assess compliance in clinical trials, such as Xhale® or AiCure®. Interestingly, one expert noted that the FDA, even with blood level data, does not allow non-adherent patients from being excluded from the data analysis. However, this information may be crucial for the analysis of the results. As some of the other points, this is relevant for the development of future protocols to assess depressed patients longitudinally in order to identify TRD/PRD patients, or to improve the clinical trial protocols for TRD/PRD, and thus we will capture the different points of view in the document.

**7) How long do you think a treatment trial must last before considering it a failure? At least 4 weeks? At least 8 weeks? More?**

Here again, we have no clear consensus. The most frequent answer is four weeks minimum (n=10). Yet, we have 5 experts each for six and eight weeks. Some views also suggest that two weeks can be enough to predict non-response to treatment. As many experts outlined, this point is strictly linked to others we will discuss, such as dosage, type of treatment, and measures of outcomes. This important issue will be discussed in *Session 3*, together with the minimal doses of antidepressant.

**8) Which antidepressants history scale or staging model would you use to define PRD or TRD?**

Many staging models have been chosen as the preferred ones, including Thase and Rush and Massachusetts General Hospital. The largest consensus emerged on the Maudsley Staging Model (n=10), probably because it best captures the dimensional nature of TRD. Some authors highlighted the need of a new approach, potentially dimensional, multi-scale and system biology oriented, which also might add further dimensions such as genetics and neurocircuitries. This topic will cut across *Session 1* and also the *Future Research Session*, and will inform the recommendation for future longitudinal studies and clinical trials.

**9) Which depressive symptoms scales and score would you use to define PRD or TRD in someone who is currently on antidepressants? Should the core symptoms scales (like HDRS6 and the MADRS6) be used instead of the full scales?**

As expected, the favoured instruments were the classic clinician-administered HAMD-17 (n=7) and MADRS-10 (n=11). Another useful tool, chosen by several experts, was the self-reported QIDS-SR (n=7). Around 40% of responders agreed with the idea of using core symptoms scales, while the other 60% was against. An interesting point is that, particularly in clinical trials, full scales (such as the HAMD-27) allow a better identification of a wide range of symptoms, leaving open the possibility to sub-classify results in different clusters, including core symptoms. Finally, also on this point some authors argued that we need to think more out of the box. All scales are too non-specific and pretend as if they are measuring some general construct, when indeed they are likely capturing small pieces of multiple constructs. Hence, the possibility to try an alternative dimensional strategy to define the symptom groups. As the point above, this debate is captured both in *Session 1* and in the *Future research Session,* and will inform the recommendation for future longitudinal studies and clinical trials.

**10) Should the criteria for full/partial response and for remission (for example, 50% drop in score or HAMD<7) be relaxed in regulatory clinical trial for PRD-TRD, to allow for the fact that these patients are unlikely to show full improvement even with new medications?**

The majority of experts agreed we do not need to relax criteria for response or remission (n=18). Interestingly, it emerged it may be useful to operationalise criteria for partial response, define something that is a lesser response or remission (for example 25-30% reduction); this specific issue will be discussed in *Session 1* for the definition of PRD.

**11) Should the definition of TRD for regulatory purpose of clinical trials include failure to augmentation/combination of antidepressants? What about failure to psychotherapeutic interventions or brain stimulation? Or should patients who have failed at these strategies be excluded from PRD-TRD regulatory trials, to minimize the variability of the study population and maximizing the chances of identify a therapeutic effect?**

This was the most open question we had in our document, and summarizing the answers was not so easy. The greater consensus we have is in favour of the inclusion of augmentation strategies in the definition of TRD, that is, patients who have not responded to augmentation should *not* be excluded from clinical trials, even though we have some opinions against. We have a majority also regarding brain stimulation techniques, with most of the experts being against its inclusion indicating that patients who failed to BS *should* be excluded for clinical trials. Psychotherapy was the tricky one, as some patients may prefer to start with psychotherapy and if they fail, they go to antidepressant. Would such patients be considered TRD? Responses were 50-50 split. Some argued that failure to psychotherapeutic interventions should be regarded as equivalent to failure to pharmacotherapy, if the interventions have been carried out as state-of-the art cognitive behavioural therapy (CBT) or psychodynamic therapy, interpersonal therapy (IPT), or cognitive behavioural analysis system of psychotherapy (CBASP). Others have expressed the opposite opinion, that is, against its inclusion in the definition, because of conflicting evidence of its efficacy and also because the failure to psychotherapy does not imply a greater resistance to pharmacological treatments. Some have argued that patients who have failed to psychotherapy are ‘more’ treatment-resistant and thus should not be included, while others that this would be a mistake as it would assume that there is some threshold that defines patients who are not going to respond to anything. One interesting point is that these patients who have failed to psychotherapy are also going to have a much lower response to placebo, and are, therefore, much more likely to show drug-placebo differences, which is very important as placebo response is the bane of these trials. This kind of patients should not be required, but also should not be systematically excluded. This debate may come up in the discussion during most Sessions.
